# β-Catenin Functions Pleiotropically in Differentiation and Tumorigenesis in Mouse Embryo-Derived Stem Cells

**DOI:** 10.1371/journal.pone.0063265

**Published:** 2013-05-14

**Authors:** Noriko Okumura, Hidenori Akutsu, Tohru Sugawara, Takumi Miura, Youki Takezawa, Akihiro Hosoda, Keiichi Yoshida, Justin K. Ichida, Mitsutoshi Yamada, Toshio Hamatani, Naoaki Kuji, Kenji Miyado, Yasunori Yoshimura, Akihiro Umezawa

**Affiliations:** 1 Department of Reproductive Biology, National Center for Child Health and Development, Tokyo, Japan; 2 Department of Obstetrics and Gynecology, Keio University School of Medicine, Tokyo, Japan; 3 Department of Stem Cell and Regenerative Biology, Harvard University, Cambridge, Massachusetts, United States of America; National Cancer Center, Japan

## Abstract

The canonical Wnt/β-catenin signaling pathway plays a crucial role in the maintenance of the balance between proliferation and differentiation throughout embryogenesis and tissue homeostasis. β-Catenin, encoded by the *Ctnnb1* gene, mediates an intracellular signaling cascade activated by Wnt. It also plays an important role in the maintenance of various types of stem cells including adult stem cells and cancer stem cells. However, it is unclear if β-catenin is required for the derivation of mouse embryo-derived stem cells. Here, we established β-catenin-deficient (β-cat^Δ/Δ^) mouse embryo-derived stem cells and showed that β-catenin is not essential for acquiring self-renewal potential in the derivation of mouse embryonic stem cells (ESCs). However, teratomas formed from embryo-derived β-cat^Δ/Δ^ ESCs were immature germ cell tumors without multilineage differentiated cell types. Re-expression of functional β-catenin eliminated their neoplastic, transformed phenotype and restored pluripotency, thereby rescuing the mutant ESCs. Our findings demonstrate that β-catenin has pleiotropic effects in ESCs; it is required for the differentiation of ESCs and prevents them from acquiring tumorigenic character. These results highlight β-catenin as the gatekeeper in differentiation and tumorigenesis in ESCs.

## Introduction

The Wnt/β-catenin signaling pathway is an evolutionarily conserved signal transduction cascade and functions during early development to regulate body axis specification, germ layer formation and organogenesis [1]. It is not surprising that mutations of the Wnt pathway components are associated with many hereditary disorders, cancer, and other diseases [2]. In preimplantation embryo development, fertilized oocytes go through a series of cleavage divisions which lead to blastocyst formation. The body axes and germ layers in mammalian embryos are established after implantation and Wnt/β-catenin signaling plays an important role in the establishment of the basic body plan in mouse embryos [1]. Embryonic stem cells (ESCs) are derived from the inner cell mass (ICM) of the mammalian blastocyst and are capable of proliferating indefinitely in culture while maintaining an undifferentiated pluripotent state [3], [4]. Wnt/β-Catenin signaling has a dominant role in the *in vitro* maintenance of both mouse and human ESCs [5]. However, it is unclear how the known function of Wnt signaling in early embryonic development relates to its regulation of pluripotency and lineage decisions in pluripotent stem cells.

β-Catenin is an essential component of the Wnt/β-catenin signaling pathway. In the absence of a Wnt signal, β-catenin is rapidly degraded by a multi-protein complex including Axin, adenomatous polyposis coli (APC) and glycogen synthase kinase 3 (GSK3). Wnt proteins stimulate signaling through various frizzled receptors, low density lipoprotein receptor-related protein 5/6 (LRP 5/6) co-receptors and disheveled to inactivate the β-catenin-degradation complex, and the stabilized β-catenin associates with transcription factors of the Tcf (T-cell factor)/Lef (lymphoid enhancer factor) family resulting in the transcriptional activation of target genes [1], [5].

Previous studies based on both loss- and gain-of-function mutations of β-catenin in mice have established the importance of the role of Wnt/β-catenin signaling in the regulation of the vertebral axis, germ layer specification and tumorigenesis [6]. Elegant studies have genetically demonstrated that β-catenin is indispensable for differentiation, but it is not required for self-renewal of mouse ESCs [7], [8]. The *Ctnnb1* deficient mouse ESC lines were established by deleting functional exons in cultured mouse ESC lines, and it still remains to be determined whether β-catenin plays an important role in the transformation of preimplantation embryos into pluripotent stem cells.

Our recent work demonstrated that β-catenin plays a role in sperm-oocyte membrane adhesion and the transition of the membrane upon mouse fertilization, using homogenous defects of β-catenin in both oocyte and sperm cells [9]. Here, we report the generation of novel Ctnnb1-deficient mouse embryo-derived stem cells (β-cat^Δ/Δ^ mESCs) with the deletion of exons 2–6 using mice containing the loss-of-function mutation in β-catenin to produce mutant oocyte and sperm through the germ cell-specific Cre-loxP system. β-cat^Δ/Δ^ mESCs are a material which can be used to address the role of β-catenin in the acquisition of pluripotency including self-renewal and differentiation from preimplantation embryos. It is also noteworthy that β-cat^Δ/Δ^ mESCs lack the residual β-catenin of either maternal or paternal origin. Our results show that β-catenin is not essential for acquiring and maintaining proliferative self-renewal, but that it is required for normal multilineage differentiation. Interestingly, these embryo-derived β-cat^Δ/Δ^ mESCs contributed to oncogenic germ cell tumors in a teratoma assay. These results were corroborated by our rescued β-cat^Δ/Δ^ mESCs experiments. Our findings show that β-catenin plays an important role in multilineage differentiation and tumorigenesis in germ cell tumors. Our embryo-derived β-cat^Δ/Δ^ mESCs also provide a novel research tool for immature germ cell tumors.

## Materials and Methods

### Generation of Completely β-catenin*-*deficient Embryos

To produce oocytes with a single gene deleted, floxed mutant mice on a C57BL/6J background with exon 2–6 flanked by loxP on the β-catenin gene (*Ctnnb1*) [10] were crossed with transgenic (Tg) mice on a C57BL/6J background expressing cre-recombinase in an oocyte-specific manner under the control of oocyte-specific zona pellucida glycoprotein 3 (ZP3) promoter (*Tg^ZP3−cre^*), which was kindly provided by Dr. Barbara B. Knowles (the Jackson Laboratory, ME, USA). The F1 offspring, β-catenin*^floxed/floxed^Tg^ZP3−cre^* (β-cat^fl/Δ^), were propagated through brother-sister mating. The presence of the cre-recombinase gene in these offspring was detected by polymerase chain reaction (PCR) analysis using the following set of primers: Cre-S [tgatgaggttcgcaagaacc; nucleotide no. 170 to 189 (GenBank Accession no. AB449974.1)] and Cre-A [ccatgagtgaacgaacctgg; nucleotide no. 539 to 558 (GenBank Accession no. AB449974.1)]. This primer set yielded a band of 389 bp. Furthermore, to produce conditionally β-catenin-deficient sperm, β-catenin*^floxed/floxed^* (β-cat^fl/fl^) mice were mated with mice expressing cre-recombinase driven by protamine promoter [11], which was kindly provided by Dr. Masaru Okabe (Osaka University, Japan). We produced β-catenin*^floxed/floxed^Tg^protamin−cre^* male mice, and confirmed that the β-catenin gene was deleted in their epididymal sperm (data not shown). Then, to obtain completely β-catenin-deficient embryos, these two strains of mice were crossed. The β-catenin-deficient (β-cat^Δ/Δ^) fertilized eggs were collected and cultured to the blastocyst stage at 37°C and 5% CO_2_ in air.

### Mouse ESC Establishment and Cell Culture

Embryo-derived stem cells were established using mouse ESCs derivation medium of knockout DMEM (Life Technologies, CA, USA) supplemented with 15% knockout serum replacement, 1 x non-essential amino acids (NEAA), 0.05 mM 2-mercaptoethanol, 2 mM GlutaMax, 100 U/mL penicillin G, 10 mg/mL streptomycin (all from Life Technologies), 1000 U/mL of leukemia inhibitory factor (LIF) (Wako, Japan) and the MEK kinase inhibitor PD98059 (Cell Signaling Technology, Danvers, MA). The blastocyst embryos developed from β-catenin-deficient fertilized eggs were individually treated with EmbryoMax Acidic Tyrode’s Solution (Merck Millipore, Germany) to remove the zona pellucidae and plated on a feeder layer. Mouse embryonic fibroblasts (MEF) were inactivated by gamma irradiation in gelatinized culture dishes with mouse ESCs derivation medium. At 48 hours after plating, one half volume of fresh medium was added to each culture well. The medium was changed daily and inner cell mass-derived outgrowths were dislodged with a Pasteur pipette, washed in a drop of fresh mouse ESC derivation medium and incubated for 5 min in StemPro Accutase (Life Technologies) at 37°C. The clumps were gently dissociated with a Pasteur pipette and transferred onto a fresh plate of MEF [12]. β-cat^Δ/Δ^ mESCs were maintained in mESC medium which was composed of derivation medium without PD98059. β-cat^Δ/Δ^ mESCs were continuously passaged every 2–3 days with 5- to 10-fold dilution by Accutase digestion. For serum-free and feeder-free cultures, mESCs were cultured in N2 and B27 medium (Life Technologies) supplemented with 1,000 U/ml LIF, 3 µM CHIR99021 (Wako) and 1 µM PD0325901 (Wako) on Matrigel-coated (BD Biosciences, San Jose, CA) dishes [13], [14]. β-cat^fl/fl^ and β-cat^fl/Δ^ mESC lines were derived directly either from β-cat^fl/fl^ or β-cat^fl/Δ^ blastocysts, respectively. The NCH4.3 ESC line (C57BL/6J) [15] and R1 ESC line (129/Sv × 129/Sv-CP) [16] were used as additional controls. The R1 ES cell line was generously provided by Dr. Andras Nagy (Mount Sinai Hospital, Toronto, Canada). F9 embryonal carcinoma (EC) cells were also used as a control in quantitative PCR array experiments. F9 EC cells were grown in a monolayer culture in DMEM (Life Technologies) containing 10% fetal bovine serum (FBS; Hyclone, Thermo Fisher Scientific Inc., UT, USA), 2 mM GlutaMax, 100 U/mL penicillin G and 10 mg/mL streptomycin (all from Life Technologies).

To detect different combinations of fl/fl, fl/Δ and Δ/Δ β-catenin alleles, the genomic PCR was performed using the following primer: (aaggtagagtgatgaaagttgtt, caccatgtcctctgtctattc and tacactattgaatcacagggactt). The resulting products of 221, 324 and 500 bp corresponded to the wild-type allele, the floxed allele and the deleted allele, respectively [9], [10].

### Differentiation Assays *in vitro* and *in vivo*

For induction of differentiation *in vitro*, 1×10^4^ feeder-independent mESCs were plated onto each well of 96-well plate with a low attachment surface (Lipidure; NOF corporation, Japan) and cultured in differentiation medium containing Knockout DMEM (Life technologies) with 20% FBS (Hyclone, Thermo Fisher Scientific), 1×NEAA, 2 mM GlutaMax, 100 U/mL penicillin G and 10 mg/mL streptomycin (all from Life Technologies), to generate embryoid bodies (EBs).

For the tumor formation assay, NCH4.3, β-cat^fl/fl^ (fl/fl#1 and 2), β-cat^fl/Δ^ (fl/Δ#2 and 3), β-catenin-reintroduced β-cat^Δ/Δ^ and β-cat^Δ/Δ^ (Δ/Δ#1, 2, 6 and 8) mESCs lines were suspended at 5×10^6^ cells/ml in mESC medium, and 200 µl of the cell suspension (1×10^6^ cells) were injected subcutaneously into the dorsal flank of nude mice (Clea Japan, Japan) using a 25-G needle. Three to four weeks after injection, tumors were surgically dissected from the mice. Samples were fixed in phosphate-buffered saline (PBS) containing 4% formaldehyde and embedded in paraffin and subjected to histological examination with hematoxylin and eosin (HE) staining. Small samples of the tissue samples were also collected for RNA isolation.

### Construction of β-catenin Constitutively Expression Plasmid

The β-catenin expression vector was constructed as follows. The mCherry was amplified by PCR with primer pairs 5′-BstBI-mCherry (ttcgaaatggtgagcaagggcgagga) and mCherry-Bam-3′ (ggatccttacttgtacagctcgtcca) to add BstBI and BamHI sites to the 5′- and 3′-sites of mCherry (Takara Bio, Japan), and cloned into pCR2.1-TOPO vector (Life Technologies), to create TOPO-mCherry. This mCherry fragment was excised from TOPO-mCherry by *Bst*BI and *Bam*HI digestion, and inserted into the BstBI and BamHI site of pIREShyg3 vector (Takara Bio), to create pCMV-mCherry-hyg. To add the 2A peptide sequence to the 3′-end, mouse β-catenin was amplified by PCR with primer pairs 5′-Nhe-Koz-Catnb (gctagcaccatggctactcaagctgacctgatggagttg) and Catnb-T2A-Cla-3′ (atcgattgggccaggattctcctcgacgtcaccgcatgttagcagacttcctctgccctctccggagcccaggtcagtatcaaaccaggccagc) and cloned into pCR2.1-TOPO vector, to create TOPO-Catnb-2A. This β-catenin-2A fragment was excised from TOPO-Catnb-2A by *Nhe*I and *Cla*I digestion, and inserted into the NheI and BstBI sites of pCMV-mCherry-hyg vector, to create pCMV-Catnb-2A-mCherry-hyg. To generate a piggyBac vector carrying a CAG promoter–driven β-catenin-2A-mCherry, β-catenin-2A-mCherry fragment was first amplified by PCR with primer pairs of 5′-Xho-Koz-Catnb (ctcgagaccatggctactcaagctgacctg) and Catnb-Bam-Cla-3′ (atcgatggatccttacttgtacagctcgtcc), then cloned into pCR2.1-TOPO vector, to create TOPO-Catnb-2A-mCherry. This β-catenin-2A-mCherry fragment was excised from TOPO-Catnb-2A-mCherry by *Xho*I and *Cla*I digestion, and inserted into the XhoI and ClaI sites of the pPB-CAG-EBNXN vector, kindly gifted by Dr. A Bradley (Wellcome Trust Sanger Institute, UK) [17], to create pPB-CAG-Catnb-2A-mCherry. All PCR-amplified fragments were verified by sequencing.

### Transfection

β-cat^Δ/Δ^ mESCs were washed with PBS once, detached by Accutase and suspended in mESC derivation medium. Cells were dissociated into a single-cell suspension by vigorous pipetting and counted. Pellets of 2×10^6^ cells were made and mixed with 1 µg of the β-catenin expression vector and 1 µg of the hyperactive piggyBac transposase expression vector (pCMV-hyPBase), kindly gifted by Dr. A Bradley, in 100 µl of mouse ES cell nucleofector solution (Lonza, Swiss). The cell suspension was transferred to a cuvette and electroporated using an Amaxa Nucleofector device (Lonza) with program A30 following the manufacturer’s protocol. The electroporated cells were plated onto 60 mm dishes on MEF in mESC medium. The mCherry-positive colonies were picked up and the clonal lines were maintained as rescued β-cat^Δ/Δ^ mESC (res-β-cat^Δ/Δ^ mESC).

### Karyotypic Analysis

Chromosome karyotyping was carried out at the Nihon Gene Research Laboratories, Miyagi, Japan).

### Alkaline Phosphatase Staining and Immunocytochemistry

Alkaline phosphatase (ALP) staining for mouse ESCs was carried out using an alkaline phosphatase kit (Dako, Denmark) according to the manufacturer’s protocol. Immunohistochemistry was carried out on mouse ESCs or EBs seeded on gelatin covered 35 mm glass coverslips (IWAKI, Japan) after fixation with 4% paraformaldehyde, followed by permeabilization in PBS with 0.5% Triton-X and blocking with PBS containing 5% normal serum appropriate for each antibody. Incubation with primary antibodies against Oct3/4 (sc-5792; Santa Cruz Biotechnology, Ca), Nanog (rcab0001P; ReproCell, Japan), Sox2 (ab5603; Merck Millipore, Germany), E-cadherin (M108; Takara Bio), β-catenin (C7207; Sigma-Aldrich, MO), α-catenin (C2081; Sigma-Aldrich), Tuj-1 (G7121;Promegam, WI), Desmin (D1033; Sigma-Aldrich), Afp (MAB1368; R&D Systems, MN) and Plakoglobin (610253; BD Biosciences) was carried out overnight at 4°C. Incubation with corresponding secondary anti-mouse, anti-rabbit and anti-goat antibodies coupled with Alexa 488 or Alexa 546 (BD Biosciences) containing 4′,6-diamidino-2-phenylindole (DAPI) for nuclei staining was carried out for one hour at room temperature in the dark in PBS containing 0.1% bovine serum albumin. Staining was observed under a LSM 720 laser scanning confocal microscope.

Tumor samples were fixed in PBS containing 4% formaldehyde and embedded in paraffin. Sections were stained with the following primary antibodies: anti-keratin5/8 (sc-70928; Santa Cruz Biotechnology), Desmin (D1033; Sigma-Aldrich), NeuN (MAB377; Merck Millipore), Oct3/4 (sc-5279; Santa Cruz Biotechnology), pan- Cytokeratin (cytokeratin 5, 6 and 8) (ab6401; abcam, UK), Sall4 (H00057167 1:100; Abnova, Taiwan), β-catenin (C7207; Sigma-Aldrich).

### Quantitative RT-PCR Analyses and Genotyping

RNA was isolated using the RNeasy Mini Kit (QIAGEN, Germany). Single strand complementary DNA was synthesized from 1–2 µg of total RNA in 20 µl of reaction mixture containing oligo-dT or random primers using the Superscript III first strand cDNA synthesis system (Life Technologies). We used two types of chemistries for the quantitative reverse transcription polymerase chain reaction (qRT-PCR); TaqMan probe-based [TaqMan Array Mouse Stem Cell Pluripotency Panel (P/N 4385363): Life Technologies] and SYBR Green-based (Platinum SYBR Green qPCR SuperMix-UDG: Life Technologies) systems. Transcript levels were determined using the ABI PRISM Sequence Detection System 7900HT (Life Technologies). All qRT-PCRs using SYBR Green were carried out in triplicate, and relative quantification was carried out using Gapdh as a reference gene. Actb, Gapdh and Eef1a1 were used as endogenous controls when we analyzed gene expression using the TaqMan Array. Hierarchical clustering analyses were performed using Ct values for gene expression data with MEV v4.8 statistical analysis software. The primers used were as follow: Oct3/4, F tgttcccgtcactgctctgg and R ttgccttggctcacagcatc; Nanog, F cctgattcttctaccagtccca and R ggcctgagagaacacagtcc; Lefty1, F gctacaacacagccatgcca and R cctcttttgcctccggagag; Afp, F ccatcacctttacccagtttgt and R cccatcgccagagtttttctt; T, F ctcggattcacatcgtgagag and R aaggctttagcaaatgggttgta; NeuroD1, F acagacgctctgcaaaggttt and R ggactggtaggagtagggatg; Ctnnb1, F ctcaccaccgcgagggcttg and R gcagtccaccagctaggcgc; Tcf3, F gacagaagtggaatttgtgtccg and R agtgcctgctactttctacgat; Sox2, F gcggagtggaaacttttgtcc and R cgggaagcgtgtacttatcctt; Klf4, F aacctttcacactgtcttcccacg and R cccttggactcttcctttctcctg; Axin2, F gcaggagcctcacccttc and R tgccagtttctttggctctt; Sox17, F agccatttcctccgtggtgt and R aacactgcttctggccctcag; Nestin, F tgcatttccttgggataccag and R cttcagaaaggctgtcacaggag; Gapdh, F tgcgacttcaacagcaactc and R cttgctcagtgtccttgctg.

### Western Blot Analysis

Western blots were carried out as described by Ishii et al. with slight modification [18]. Western blots were carried out using the following antibodies: β-catenin (C-7738; Sigma-Aldrich), Oct3/4 (sc-5279; Santa Cruz Biotechnology), Nanog (REC-RCAB0002PF; ReproCell) and β-actin (F3777; Sigma-Aldrich).

### Blastocyst Injections to Produce Chimeras

To visualize the *in vivo* contribution of β-cat^Δ/Δ^ mESCs, β-cat^Δ/Δ^ mESCs, were transfected with the constitutive enhanced green fluorescence protein (EGFP) expression vector (pCAG-EGFP) [19]. After screening, we isolated the GFP-positive β-cat^Δ/Δ^ mESC (EGFP-β-cat^Δ/Δ^ mESC) line, which was continuously cultured on feeder layers in mESC derivation medium with LIF. EGFP-β-cat^Δ/Δ^ mESCs were injected into blastocysts of ICR mice, then transferred to the uteri of pseudopregnant ICR mice, as described previously [15], [20]. Res-β-cat^Δ/Δ^ mESCs with mCherry fluorescence were used for blastocyst injections as donor cells. Embryos were dissected either on embryonic day (E) 10.5 or 12.5 and the contribution of mESCs to embryos was assessed using a fluorescence stereomicroscope (MVX10, OLYMPUS, Japan).

### Animal Studies

All animal experiments were performed according to protocols approved by the Institutional Animal Care and Use Committee of the National Research Institute for Child Health and Development (NRICHD, Permit Number: A2006-009). Mice were housed in plastic cages lined with soft wood chips. The cages were kept in a conventional, air-conditioned mouse room under a 12 h light/dark cycle and the mice received food and water *ad libitum*. All the transgenic mice were monitored for signs of suffering including tumor formation and weakening at least every two days. All surgery was performed under isoflurane anesthesia. Mice were euthanized by carbon dioxide inhalation at humane endpoints or before the collection of embryos and tissue samples as recommended by the American Veterinary Medical Association Panel on Euthanasia. All experiments were subject to the 3 R consideration (refine, reduce and replace) and all efforts were made to minimize animal suffering, and to reduce the number of animals used.

## Results

### Characterization of β-catenin Deficient Mouse Embryo-derived Stem Cells

**Figure 1:**
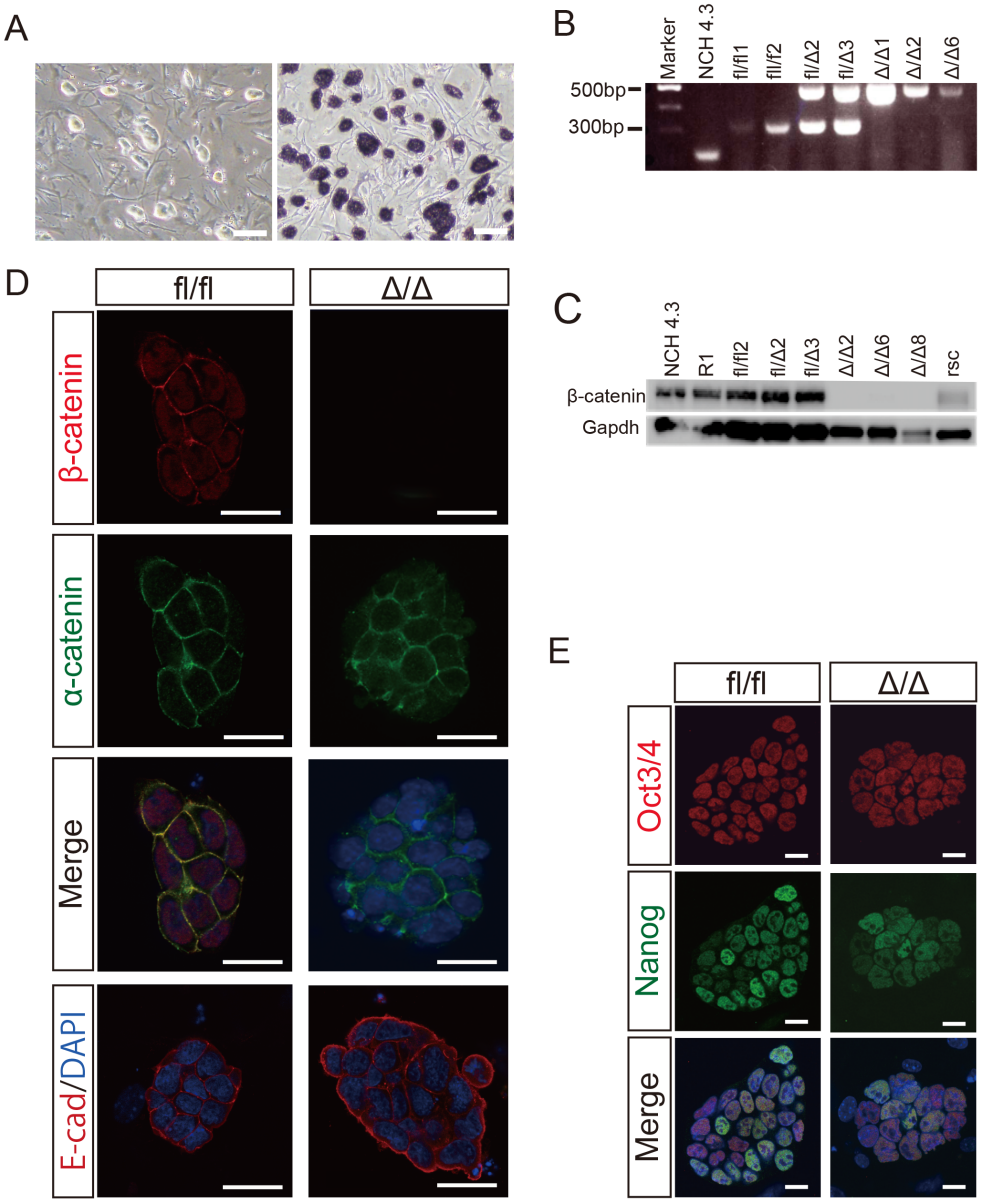
Characterization of embryo-derived β-cat^Δ/Δ^ mESCs. (A): Morphological appearance of β-cat^Δ/Δ^ mESCs is shown in the left panel. ALP staining of β-cat^Δ/Δ^ mESCs is shown in the right panel. Scale bars are 150 µm. (B): Electrophoretic analysis of the genotyping PCR for wild-type (NCH4.3), β-cat^fl/fl^ (fl/fl1 and fl/fl2), β-cat^fl/Δ^ (fl/Δ2 and fl/Δ3) and β-cat^Δ/Δ^ (Δ/Δ1, Δ/Δ2 and Δ/Δ6) mESCs. (C): Western blots of β-catenin and Gapdh in wild-type (NCH4.3 and R1), β-cat^fl/fl^ (fl/fl2), β-cat^fl/Δ^ (fl/Δ2 and fl/Δ3), β-cat^Δ/Δ^ (Δ/Δ2, Δ/Δ6 and Δ/Δ8) and β-catenin rescued β-cat^Δ/Δ^ mESCs (rsc). (D and E): Immunofluorescence staining for β-catenin (red), α-catenin (green), E-cadherin (red) and Oct3/4 (red), Nanog (green) of β-cat^fl/fl^ and β-cat^Δ/Δ^ mESC colonies as observed under confocal microscopy. Nuclei are stained for DAPI (blue). Scale bars in (D) and (E) are 20 µm.

We have previously reported the role of β-catenin in sperm-oocyte adhesion using transgenic mice containing oocyte- and sperm-specific cre-recombinase [9]. Using these mouse models, we produced β-catenin-deficient blastocysts by collecting two-cell embryos and developing them *in vitro*. The success rate for achieving blastocyst development was around 80%. We established 12 independent stem cell lines from β-catenin-deficient blastocysts and referred to them as β-cat^Δ/Δ^ mESCs (Δ/Δ1–12) (Figure 1A). PCR genotyping of the β-cat^Δ/Δ^ mESCs verified the deletion of exon 2–6 of β-catenin (Figure 1B). In addition, western blot analysis confirmed the absence of β-catenin protein in all the β-cat^Δ/Δ^ mESCs lines tested (Figure 1C). Furthermore, immunocytochemistry confirmed the absence of β-catenin protein, in contrast to wild-type mESCs, which showed staining of β-catenin in both the nucleus and cell membrane, whereas α-catenin and E-cadherin staining and distribution were similar to β-cat^fl/fl^ mESCs (Figure 1D). Plakoglobin was firmly stained in β-cat^Δ/Δ^ mESCs (Figure S7). β-cat^Δ/Δ^ mESCs showed similar proliferation behavior and morphological appearance to β-cat^fl/fl^ mESCs. This adaptation of β-cat^Δ/Δ^ mESCs is likely due to compensatory upregulation of plakoglobin, which can substitute for β-catenin in cell-adhesion junctions of mESCs [7], [8]. The embryos of the loss-of-function mutation of β-catenin developed to the blastocyst stage *in vitro*, but the crossbred female mice produced β-catenin-deficient embryos that died at E7.0 post-conception [9], [21], [22]. We randomly chose several β-cat^Δ/Δ^ mESCs lines for further analysis.

The β-cat^Δ/Δ^ mESCs were morphologically similar to β-cat^fl/fl^ mESCs, expressed Alkaline Phosphatase, and were karyotypically normal (Figures 1A, S1A). Pluripotency factors such as Oct3/4 and Nanog were immunocytochemically positive in the nuclei of both β-cat^fl/fl^ and β-cat^Δ/Δ^ mESCs (Figure 1E). The expression levels of pluripotent marker genes (Oct3/4, Nanog, Klf4 and Sox2) and proteins (Oct3/4 and Nanog) in β-cat^fl/fl^ and β-cat^fl/Δ^ mESCs were comparable to those of β-cat^Δ/Δ^ mESCs, as assessed by quantitative RT-PCR (qRT-PCR) analysis and western blotting (Figures S1B, S2). Multiple gene expression analysis by qRT-PCR using TaqMan Array Mouse Stem Cell Pluripotency Card showed that β-cat^Δ/Δ^ mESCs were similar to wild-type and res-β-cat^Δ/Δ^ mESCs (Figure S5A).

To further confirm the functional deficiency of β-catenin in β-cat^Δ/Δ^ mESCs in culture, we expanded β-cat^Δ/Δ^ mESCs under serum- and feeder-free conditions using the 2i+LIF system with mitogen-activated kinase kinase (MEK) inhibitor, PD0325901, and GSK3β inhibitor, CHIR99021 [13], [14]. β-cat^Δ/Δ^, under the serum- and feeder-free conditions using the 2i+LIF system, β-cat^fl/Δ^, and β-cat^fl/fl^ mESCs showed a similar rate of cell proliferation (Figure S1C). We could maintain β-cat^Δ/Δ^ mESCs under the 2i+LIF system as β-cat^fl/fl^ mESCs, although partial colonies exhibited a different morphology than previously reported [7] (Figure S3A). We examined Axin2 expression levels in β-cat^fl/fl^ and β-cat^Δ/Δ^ mESC (Figure S3B) using qRT-PCR. Exposure to GSK3β inhibitor (2i+LIF) increased the expression of Axin2 in β-cat^fl/fl^ mESCs, while down-regulating Axin2 in β-cat^Δ/Δ^ mESCs (Figure S3B). Accordingly, the endogenous Wnt/β-catenin/Tcf/Lef-regulated gene Axin2 was not up-regulated in our β-cat^Δ/Δ^ mESCs (Figure S3B). Thus, we further confirmed that β-cat^Δ/Δ^ mESCs are defective in the Tcf/Lef-mediated signaling of the canonical Wnt/β-catenin pathway.

Overall, we successfully derived stem cells that were capable of self-renewal directly from β-catenin-deficient preimplantation embryos. These β-cat^Δ/Δ^ mESCs can maintain clonogenicity in culture either with or without feeders in serum-free conditions. Thus β-catenin is not required for self-renewal in mESCs.

### Differentiation Defects of Embryo-derived β-cat^Δ/Δ^ mESCs in EBs and Blastocyst Complementation Assays

**Figure 2:**
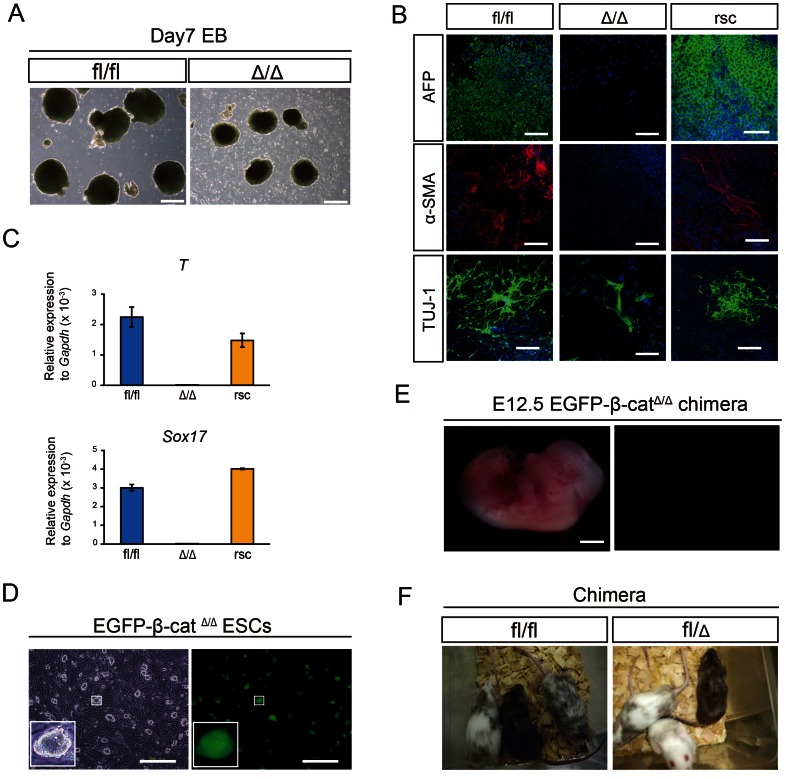
Differentiation potential of embryo-derived β-cat^Δ/Δ^ mESCs. (A): Phase contrast pictures of EBs derived from β-cat^fl/fl^ and β-cat^Δ/Δ^ mESCs on day 7 of differentiation. Scale bars are 500 µm. (B): Immunofluorescence staining for Afp (green), α-SMA (red) and Tuj-1 (green) of β-cat^fl/fl^, β-cat^Δ/Δ^ and β-catenin rescued β-cat^Δ/Δ^ mESCs on day 14 of differentiation. Nuclei are stained for DAPI (blue). Scale bars are 200 µm. (C): Expression levels of Sox17 and Brachyury(T) relative to Gapdh in β-cat^fl/fl^ (blue bar), β-cat^Δ/Δ^ (light blue bar) and β-catenin rescued β-cat^Δ/Δ^ (yellow bar) mESCs on day 6 of differentiation. (D): Fluorescence microscopic images of EGFP-β-cat^Δ/Δ^ mESC colonies. They constitutively expressed EGFP. Scale bars are 500 µm. (E): Fluorescent stereomicroscopic image of an embryo on E12.5 generated from blastocysts injected with EGFP-β-cat^Δ/Δ^ mESCs. The contribution of β-cat^Δ/Δ^ mESCs were barely detected anywhere in the whole body. Scale bars are 2 mm. (F): Chimeras generated by injection of β-cat^fl/fl^ and β-cat^fl/Δ^ mESCs into ICR host blastocysts. Offspring coat color demonstrates high contribution chimeras.

We sought to determine whether β-cat^Δ/Δ^ mESCs have impaired differentiation potential compared to wild-type mESCs. We created EBs in differentiation medium without LIF from β-cat^fl/fl^, β-cat^Δ/Δ^ and β-catenin-reintroduced β-cat^Δ/Δ^ mESCs. β-cat^Δ/Δ^ EBs were smaller in size compared to β-cat^fl/fl^ EBs (Figure 2A). The extent of the defect in differentiation in β-cat^Δ/Δ^ EBs was assessed by immunofluorescence staining for differentiation markers after 14 days. Multiple germ layer formation was severely impaired in β-cat^Δ/Δ^ EBs, but not β-cat^fl/fl^ EBs (Figure 2B). At the transcription level, early differentiation markers such as Sox17, and brachyury (T) were detectable on day 6 of EB induction in β-cat^fl/fl^ EBs, but transcripts of these genes were expressed at much lower level in β-cat^Δ/Δ^ EBs (Figure 2C). Our results of extremely down-regulated expression of brachyury (T) in β-cat^Δ/Δ^ EBs are in agreement with previous findings that brachyury (T) is a direct transcriptional target of β-catenin [23].

To assess the contribution of embryo-derived β-cat^Δ/Δ^ mESCs *in vivo*, we performed a chimera assay by blastocyst injection using EGFP-β-cat^Δ/Δ^, β-cat^fl/fl^ and β-cat^fl/Δ^ mESC lines. EGFP-β-cat^Δ/Δ^ mESCs were stably maintained with constitutive EGFP expression (Figure 2D). Different lines of EGFP β-cat^Δ/Δ^ mESCs were injected into blastocysts and transferred into pseudopregnant mice and the thirty-eight resulting live offspring exhibited no chimeric phenotype. In contrast, the live pups from β-cat^fl/Δ^ and β-cat^fl/fl^ mESC lines showed high concentrations of the chimeric phenotype (Figure 2F). To further evaluate the ability of β-cat^Δ/Δ^ mESCs to differentiate *in vivo*, EGFP-β-cat^Δ/Δ^ mESC-injected embryos were recovered on E12.5. Macro zoom fluorescence microscopy images revealed that the contribution of EGFP-β-cat^Δ/Δ^ mESCs was barely detectable in whole body embryos on E12.5 (Figure 2E), although a small contribution was rarely detected in some malformed fetuses (Figure S6). Taken together, embryo-derived β-cat^Δ/Δ^ mESCs are significantly impaired in their ability to differentiate properly *in vitro* and *in vivo*.

### Aberrant *in* *vivo* Differentiation of β-cat^Δ/Δ^ mESCs in Teratoma Assay and Subsequent Development into Germ Cell Tumors

**Figure 3:**
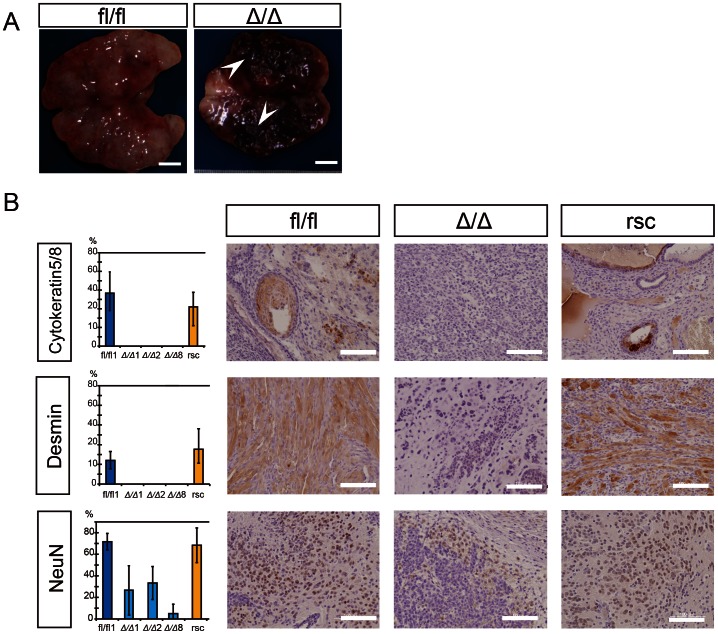
Immunohistochemical examination of tumors generated from ESCs either β-cat^Δ/Δ^ or β-cat^fl/fl^. (A): The gross image of a tumor mass derived from β-cat^fl/fl^ mESC is shown on the left and that of a tumor derived from β-cat^Δ/Δ^ mESCs is shown on the right. β-cat^Δ/Δ^ tumor mass was characterized by extensive intra-tumoral hemorrhage (white arrow head). Scale bars are 5 mm. (B): Immunohistochemical staining for Cytoketratin 5/8, Desmin and Neuronal nuclear antigen (NeuN) in tumors derived from mESCs of β-cat^fl/fl^, β-cat^Δ/Δ^ or res-β-cat^Δ/Δ^. Tissue sections of β-cat^Δ/Δ^ tumors displayed high level staining only for the neuronal differentiation marker NeuN, while there was no detectable staining for Cytoketratin 5/8 or Desmin. Tissue sections of both β-cat^fl/fl^ and res-β-cat^Δ/Δ^ tumors displayed multiple differentiations as shown in three markers’ positive staining. The left bar graph shows percentages of Cytoketratin 5/8, Desmin and NeuN positive areas with standard deviation (n = 3) as the vertical axis and each tumor as the horizontal axis. Scale bars are 100 µm.

**Figure 4:**
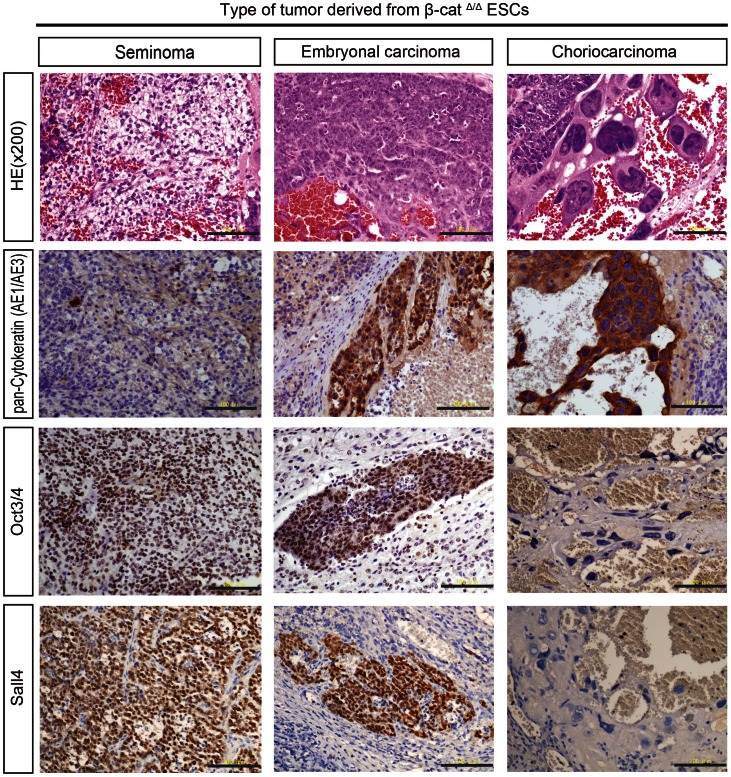
Histological and immunohistochemical examination of β-cat^Δ/Δ^ tumors. Careful examination of numerous tumor sections stained with hematoxylin and eosin (HE) from different β-cat^Δ/Δ^ mESC lines generated independently in separate animals revealed that each β-cat^Δ/Δ^ tumor contained three subtypes (seminoma, embryonal carcinoma, choriocarcinoma) of carcinomatous germ cell tumor components. The “seminoma” component is characterized by large uniform dispersed tumor cells displaying clear cytoplasm and distinct cell membrane. Histologic features of the “seminoma” were “fried-egg” appearance: central nucleus with nucleolus, clear cytoplasm and well-defined cell borders. The “embryonal carcinoma (EC)” displayed a papillary pattern, with cohesive clustered growth and marked cytologic atypia. The “choriocarcinoma” was identified by syncytiotrophoblastic giant cells with extensive hemorrhage. The syncytiotrophoblastic giant cells had huge, pleomorphic nuclei and abundant eosinophilic cytoplasm. The “seminoma” displayed very high levels of nuclear OCT4 and Sall4 immunoreactivity, but was negative for pan-cytokeratin. “EC” was strongly positive for pan-cytokeratin, Oct3/4 and Sall4. The syncytiotrophoblastic giant cells of the “choriocarcinoma” showed positive cytoplasmic staining for pan-cytokeratin, but negative nuclear immunostaining for Oct3/4 and Sall4. Scale bars are 100 µm.

Wild-type pluripotent ESCs generate benign teratomas composed of well differentiated tissues of endo-, meso- and ectodermal origin. We tested the ability of β-catenin-deficient and β-cat^fl/fl^ mESCs to form the three germ layer lineages in teratomas. After three to five weeks of subcutaneous injection, the tumors which had developed were excised and histologically analyzed. The tumors derived from β-cat^Δ/Δ^ mESCs were grossly characterized by areas of hemorrhage and necrosis, which were hardly observed in the teratomas derived from β-cat^fl/fl^ mESCs (Figure 3A). All tumors generated from independent β-cat^Δ/Δ^ mESC lines showed severe differentiation defects in histological sections (Figure 3B, middle column) and a grossly undifferentiated cancer-like appearance (Figure 4). Multiple gene expression analysis of teratoma, tumor and F9 embryonal carcinoma (EC) cells by qRT-PCR using a TaqMan Array Mouse Stem Cell Pluripotency Card revealed that teratomas derived from wild-type and res-β-cat^Δ/Δ^ mESCs were clearly separate from tumors derived from β-cat^Δ/Δ^ mESCs and F9 EC cells, whereas the gene expression pattern of undifferentiated wild-type, res-β-cat^Δ/Δ^ and β-cat^Δ/Δ^ mESCs were very similar to each other and differed only from the F9 EC cells (Figure S5B). As subtle differentiation is difficult to recognize using morphological criteria of tumors generated from β-cat^Δ/Δ^ mESC lines, we used three differentiation markers to identify the cellular types. Cytokeratin 5/8 and Desmin antibodies did not stain tumor sections of β-cat^Δ/Δ^ mESC lines, in contrast to β-cat^fl/fl^ and res-β-cat^Δ/Δ^ teratomas in which approximately 10–40% of the cells were positively stained (Figure 3B). Also, the neuron-specific nuclear protein marker, Neuronal Nuclei (NeuN) was expressed in the tumor sections of β-cat^Δ/Δ^ mESC lines, but at a much lower rate than in the teratomas of β-cat^fl/fl^ and res-β-cat^Δ/Δ^ (Figure 3B).

**Figure 5:**
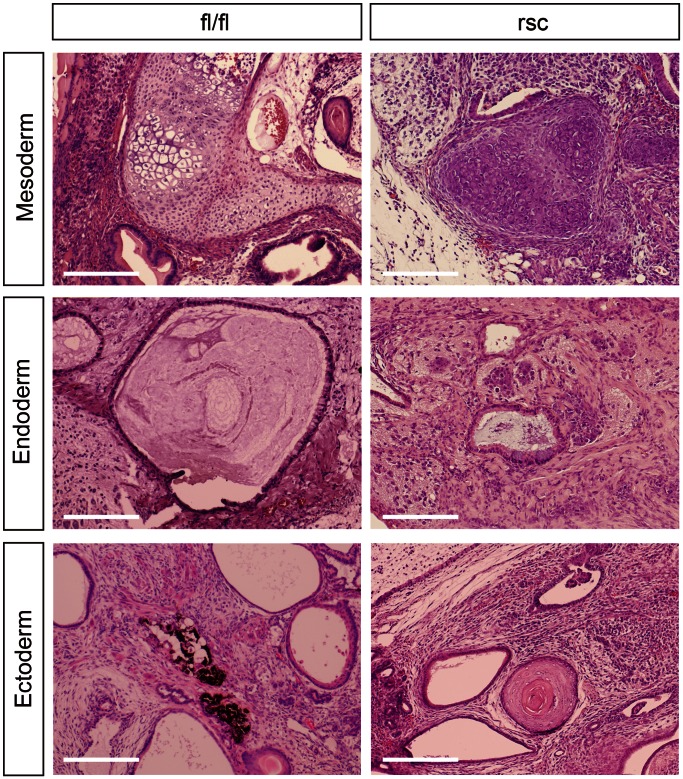
The histological tumorigenic properties of β-cat^Δ/Δ^ ESCs can be restored to wild-type ESCs. β-cat^fl/fl^ teratomas consisted of well-differentiated mesodermal tissues (bone and cartilage), endodermal tissues (glandular epithelial structures) and ectodermal tissues (pigmented cells). β-catenin-rescued β-cat^Δ/Δ^ mESCs (res-β-cat^Δ/Δ^ mESCs) gave rise to multilineage differentiated teratoma possessing mesoderm (bone and cartilage), endoderm (glandular epithelial structures) and ectoderm (epidermal tissue). Scale bars are 100 µm.

Careful observation of numerous tumor sections of different β-cat^Δ/Δ^ mESC lines generated independently in separate animals surprisingly revealed pathological carcinomatous features resembling heterogeneous mixtures of germ cell tumors (GCTs) composed of seminoma, embryonal carcinoma, and choriocarcinoma characters (Figure 4). While the well-characterized human germ cell tumors allows identification for histologic classification [24], to date, no animal model is available for analyzing testicular GCT formation and progression [25]. “Seminoma” lesions in β-cat^Δ/Δ^ tumors were histologically characterized by a composition of tubular structures lined by seminoma cells with clear cytoplasm, and well-defined cytoplasmic borders, central to marginally located nuclei. These gross appearances were similar to that of classic seminoma in humans in that they had a “fried-egg” appearance (Figure 4). “Embryonal carcinoma (EC)” lesions of the β-cat^Δ/Δ^ tumors displayed a papillary pattern, cohesive clustered growth with marked cytologic atypia, and central necrosis of the lesion (Figure 4). “Choriocarcinoma” lesions were easily identified in the β-cat^Δ/Δ^ tumor sections by the area of hemorrhage and necrosis both macro- and microscopically. They were characterized by the coexistence of smaller cells with clear cytoplasm and the much larger syncytiotrophoblastic giant cells with huge, pleomorphic nuclei and abundant eosinophilic cytoplasm (Figure 4). Testicular GCTs in human are heterogeneous and contain diverse histopathology and variable clinical course and prognosis [26]. To classify GCTs, immunomarkers are vital. Pluripotent stem cell markers such as OCT3/4, NANOG, and SALL4 are used in clinical trials as they are very sensitive and specific markers [26], [27], [28]. In our immunohistochemical analysis using specific markers of GCTs, pan-Cytokeratin (AE1/AE3) were focally positive in “EC” lesions and strongly positive in “choriocarcinoma” areas of the β-cat^Δ/Δ^ tumors, but not expressed in the “seminoma” areas. OCT3/4 is one of the most robust diagnostic markers of human testicular GCTs [25]. “Seminomas” and “ECs” lesions showed strong positive nuclear staining for Oct3/4, but “choriocarcinoma” cells were negative. SALL4 has been reported to be expressed in subtypes of GCT with high sensitivity and specificity [28], [29]. “Seminoma” and “EC” lesions showed positive staining, but “choriocarcinoma” areas of the β-cat^Δ/Δ^ tumors were negative. Notably, in all attempts, β-cat^Δ/Δ^ mESC lines generated tumors with a carcinomatous appearance, whereas wild-type counterparts gave rise to teratomas with normal differentiation patterns (Figure 5).

**Figure 6:**
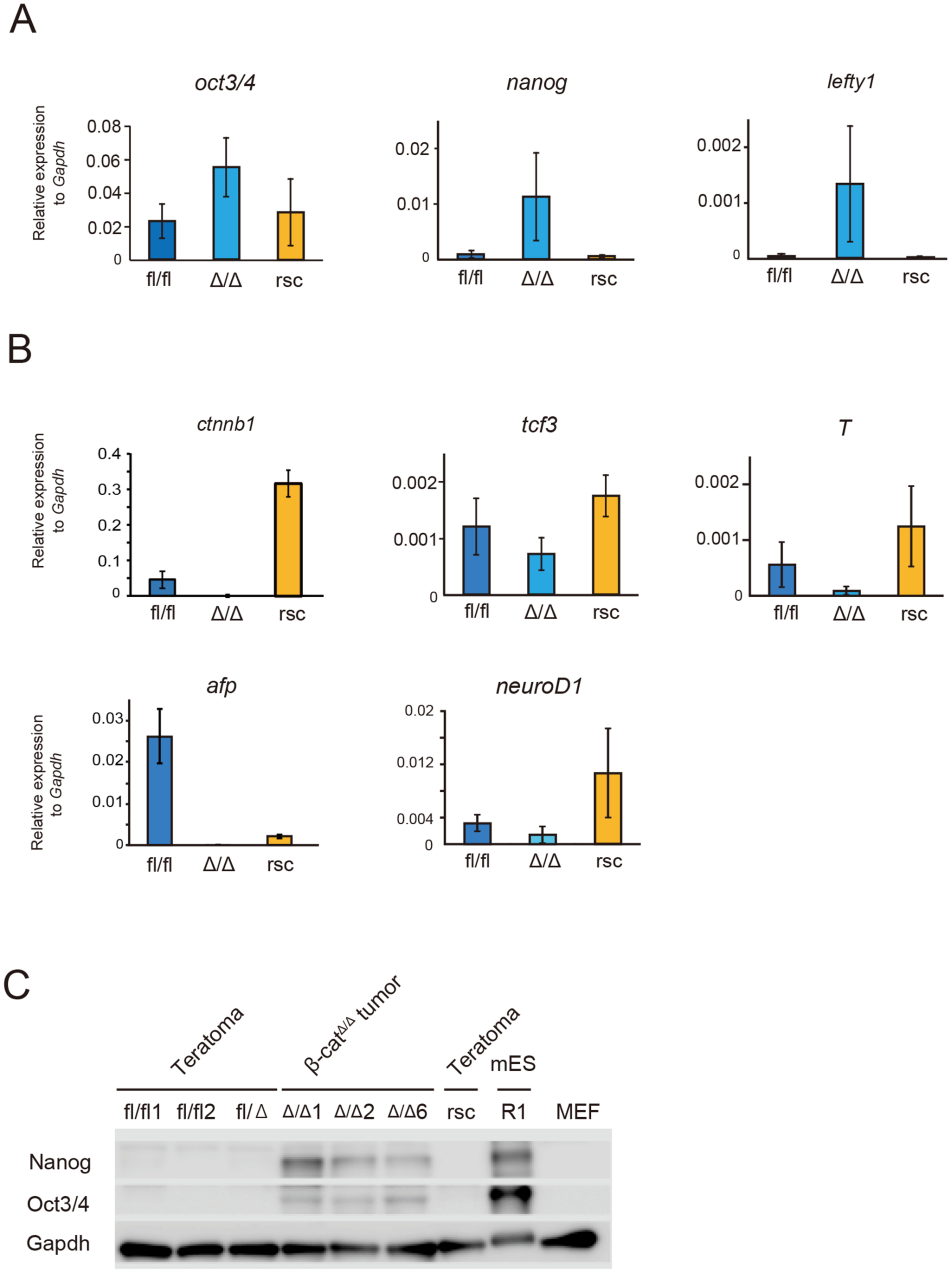
Quantitative PCR and western blot examination of wild-type ESCs derived teratomas, res-β-cat^Δ/Δ^ teratomas and β-cat^Δ/Δ^ tumors. (A and B): Expression levels of self-renewal marker genes (oct3/4, nanog, lefty1) and the downstream genes of Wnt/β-catenin signaling (Ctnnb1, tcf3, T) and early differentiation markers (afp, neuroD1, T) relative to Gapdh in β-cat^fl/fl^ (blue bar), res-β-cat^Δ/Δ^ (yellow bar) and β-cat^Δ/Δ^ (light blue bar) mESC derived teratomas or tumors. (C) Western blots of Nanog, Oct3/4 and Gapdh for teratomas derived from β-cat^fl/fl^ (fl/fl1,fl/fl2), β-cat^fl/Δ^, res-β-cat^Δ/Δ^ (rsc) and R1 mESCs, tumors derived from β-cat^Δ/Δ^ (Δ/Δ1, Δ/Δ2, Δ/Δ6) mESC. MEF was used as a control. Abbreviation: MEF, mouse embryonic fibroblast.

We sought to confirm our findings by qRT-PCR for pluripotent markers. Quantitative RT-PCR analysis was performed independently more than three times for each sample. β-cat^Δ/Δ^ tumors expressed oct3/4, nanog and lefty1 at high levels (Figure 6A). In contrast, transcript levels of oct3/4, nanog and lefty1 in β-cat^fl/fl^ and res-β-cat^Δ/Δ^ teratomas were markedly lower than those in β-cat^Δ/Δ^ tumors (Figure 6A). In addition, three germ layer differentiation markers of afp, brachyury (T) and neuro D1 were down-regulated in β-cat^Δ/Δ^ tumors, while these differentiation markers were clearly detected in β-cat^fl/fl^ and res-β-cat^Δ/Δ^ teratomas (Figure 6B). Additionally, western blot analysis showed that Nanog and Oct3/4 were detected at the protein level in β-cat^Δ/Δ^ tumors, while they were undetected in teratomas generated from β-cat^fl/fl^ mESC lines (Figure 6C).

In the clinical diagnosis of GCTs, most yolk sac tumors produce AFP, but classic seminomas, pure ECs and choriocarcinomas do not [30]. The afp gene was expressed in teratomas induced by β-cat^fl/fl^ and res-β-cat^Δ/Δ^ mESCs, but not in tumors induced by β-cat^Δ/Δ^ mESCs. Lack of afp expression in tumors induced by β-cat^Δ/Δ^ mESCs is compatible with the results of the histological analysis in that β-cat^Δ/Δ^ tumors did not contain yolk sac elements. Hence, our teratoma assay data strongly suggest that embryo-derived β-cat^Δ/Δ^ mESCs transformed into grossly undifferentiated malignant tumors composed of testicular mixed germ cell tumors including classic seminomas, embryonal carcinomas and choriocarcinomas.

### β-cat^Δ/Δ^ ESC Properties can be Rescued with a wild-type β-catenin

We generated a piggyBac vector carrying a CAG promoter–driven wild-type β-catenin-2A-mCherry for long-term stable expression to restore the differentiation deficiency of β-cat^Δ/Δ^ mESCs. We confirmed the establishment of β-catenin- expression in β-cat^Δ/Δ^ mESC (res-β-cat^Δ/Δ^ mESC) by mCherry expression (Figure S4A) and β-catenin protein (Figures 1C, S4B). β-Catenin, α-catenin and E-cadherin staining and distribution of res-β-cat^Δ/Δ^ mESCs were similar to β-cat^fl/fl^ mESCs (Figure S4B). The *in vitro* differentiation assay showed that endoderm, mesoderm, and ectoderm differentiation of res-β-cat^Δ/Δ^ mESCs were restored and were indistinguishable from β-cat^fl/fl^ mESCs (Figure 2B and 3B). In the chimera assay, we readily detected the contribution of res-β-cat^Δ/Δ^ mESCs in the body at E10.5 (Figure S4C). Together, our rescue experiment results suggest that the re-expression of β-catenin in β-cat^Δ/Δ^ mESCs is sufficient to restore them to a wild-type-like mESC identity in *in vitro* and *in vivo*. Furthermore, we sought to analyze whether re-expression of β-catenin can prevent oncogenesis of β-cat^Δ/Δ^ mESCs. Histological analysis of res-β-cat^Δ/Δ^ tumors showed well-differentiated tissues of endo-, meso- and ectodermal origin instead of the undifferentiated carcinomatous appearance of mixed GCTs (Figure 5). The expression pattern of pluripotent and differentiation markers in res-β-cat^Δ/Δ^ tumors became comparable to that in β-cat^fl/fl^ tumors (Figure 6). Tumors derived from res-β-cat^Δ/Δ^ mESCs were histopathologically diagnosed as mature teratomas, composed of well multi-differentiated tissues. Thus, embryo-derived β-cat^Δ/Δ^ mESCs lost cancerous properties and gained multiple differentiation potential after the restoration of expression of wild β-catenin. Taken together, our rescue data suggest that the restoration of expression of wild-type β-catenin in β-cat^Δ/Δ^ mESCs is sufficient to restore the wild-type-like identity of ESCs.

## Discussion

Multicellular organisms require stem cells and precise control of them to maintain tissue homeostasis, the replacement of terminally differentiated, aged or damaged cells [31]. Loss of stem cell activity and tissue homeostasis is likely linked to the emergence of diseases including cancer [32], [33]. There is considerable interest in finding ways of perpetuating the pluripotency of embryo-derived cells, and gaining this knowledge is of vital importance for many potential biomedical applications related to tissue engineering and disease modeling [31], [34]. ESCs are one of the most characterized embryo-derived pluripotent stem cells and Wnt/β-catenin signaling has been implicated in their maintenance for both mouse and human [35], [36]. The ICM of the blastocyst delaminates giving rise to a primitive ectoderm and a primitive endoderm layer, and further differentiated cells progressively lose their differentiation potential. ESCs can be derived from the pluripotent ICM of blastocysts. In addition, β-catenin does not play a crucial role during preimplantation development [37], and at the implantation stage, inactivation of the canonical Wnt/β-catenin signaling pathway blocks blastocyst competency for implantation [38]. It remains unclear whether this signaling is crucial for the acquisition of pluripotency from the ICM of β-catenin-deficient preimplantation embryos.

### Acquisition of Self-renewal Capacity, but Impaired Multilineage Differentiation after ESC Derivation without β-catenin

Previous studies failed to eliminate the effects of remnant β-catenin of maternal or paternal origin. Our conditional mutagenesis studies of β-catenin excluded remnant β-catenin in sperm and eggs, demonstrating that β-catenin is dispensable for fertilized eggs. Furthermore, β-catenin is not required for the acquisition of self-renewal potential by embryo-derived stem cells. However, β-catenin is an indispensable prerequisite for pluripotency. Embryos lacking functional β-catenin activity show a development loss following implantation at around E7.0 with defects in anterior-posterior patterning and mesoderm and definitive endoderm formation [9], . The role of β-catenin in early development remained to be elucidated because embryonic lethality is associated with the loss-of-function mutation. We observed that the *in vitro* differentiation of embryo-derived β-cat^Δ/Δ^ mESCs is impaired similarly to β-catenin-mutant embryos [21], [22]. β-Catenin-defect mESCs contribute to chimeric embryos, but the contribution ratio is quite low compared to control mESCs [7]. Previous experiments made observations at earlier times of pregnancy (E7.0 to E8.5). In contrast, our chimeric embryo studies were analyzed on E12.5 and no chimeric compartments were detected. This may have been due to the progressive loss of contribution of β-catenin-defective cells. In other words, β-catenin is required for cellular differentiation and/or viability in early embryogenesis up to E12.5. Notably, the defects of β-cat^Δ/Δ^ mESCs in differentiation *in vitro* and *in vivo* could be rescued by re-introducing functional β-catenin. Thus, β-catenin is a caretaker in both *in vitro* ESC differentiation and *in vivo* cellular differentiation during development.

### Implicating Loss-of-function Mutation in β-catenin in Testicular Germ Cell Tumorigenesis

The central player in the canonical Wnt cascade is β-catenin, a cytoplasmic protein whose stability is regulated by the destruction complex. The tumor suppressor protein, Axin acts as the scaffold of this complex as it directly interacts with all other components including β-catenin, the tumor suppressor protein, APC, and the two kinase families CK1 and GSK3 [39]. Deregulated Wnt/β-catenin signaling is associated with many human diseases, including cancer, osteoporosis, aging and degenerative disorders [39], [40], [41]. Constitutive β-catenin signaling, resulting from deficiency of the destruction complex or oncogenic β-catenin mutations that prevent its degradation, leads to excessive stem cell renewal/proliferation that predisposes cells to tumorigenesis [2], [42]. Previously, two groups of researchers independently showed that APC mutant and GSK3α/β double-mutant mESCs remain undifferentiated and possess a malignant phenotype in the teratoma assay [43], [44]. APC-mutant mESCs generated undifferentiated carcinomatous tumors with raised levels of β-catenin [43], while GSK3α/β double-mutant mESCs generated grossly undifferentiated carcinomatous tumors with bone tissues as the only differentiated structures [44]. The mechanisms underlying the oncogenesis of both the APC-mutant and GSK3α/β double-mutant mESCs is unclear. Our β-catenin loss-of-function mESCs displayed a tumorigenic phenotype with the carcinomatous appearance of mixed germ cell tumors in a teratoma assay. The tumors derived from our embryo-derived β-cat^Δ/Δ^ mESCs included a mixture of classic seminomas, embryonal carcinomas and choriocarcinomas, identifications we made based on the histological features of human carcinomas [24], [26]. All examined tumors of β-cat^Δ/Δ^ mESCs consistently expressed the pluripotency markers Oct4, Nanog and Lefty1, while β-cat^fl/fl^ mESCs failed to maintain expression of these markers. We successfully rescued the multilineage differentiation potential of β-cat^Δ/Δ^ mESCs by restoring the expression of functional β-catenin. Loss of β-Catenin can still maintain self-renewal of mESCs and sustain pluripotency markers such as Nanog and Oct3/4. The failure in repression of pluripotencial genes might correlate with formation of germ cell tumor in β-cat^Δ/Δ^ mESCs. To our knowledge, no animal model enables us to study tumorigenesis of mixed germ cell tumors. Human germ cell tumors are a heterogeneous type of neoplasm, mostly originate from germ cells [27]. The characteristics of germ cell tumors are either derived from the process of tumorigenesis or a reflection of normal embryonal development, a feature which contributes to the complexity of these tumors [25]. Embryo-derived pluripotent stem cells allow a better understanding of the biology of GCTs.

Our results reveal that β-catenin signaling guides healthy development and normal cellular differentiation, and disruption of β-catenin signaling initiates oncogenicity turning embryo-derived stem cells into mixed germ cell tumors. The re-introduction of β-catenin expression can rescue the normal pluripotency, highlighting potential clinical approaches for clinical treatments of mixed germ cell tumors. There is currently no animal model of testicular mixed germ cell tumors [25]. Therefore, β-cat^Δ/Δ^ mESCs are novel research tools for the study of tumorigenesis of testicular germ cell tumors and the function of β-catenin in cell-fate switching between tissue homeostasis and oncogenesis.

## Supporting Information

Figure S1 **Growth curve, western blotting and chromosome analysis of embryo-derived β-cat^Δ/Δ^ mESCs.** (A): The examined β-cat^Δ/Δ^ mESC lines had normal karyotypes. The β-cat^Δ/Δ^ (Δ/Δ2) mESC line showed a normal karyotype of 40XX. (B): Western blots of Nanog and Oct3/4 in wild-type (NCH4.3 and R1), β-cat^fl/fl^ (fl/fl2), β-cat^fl/Δ^ (fl/Δ2 and fl/Δ3), β-cat^Δ/Δ^ (Δ/Δ2, Δ/Δ6 and Δ/Δ8) mESCs and MEF. (C): Growth curve of β-cat^fl/fl^ (fl/fl2), β-cat^fl/Δ^ (fl/Δ3), β-cat^Δ/Δ^ (Δ/Δ6 and Δ/Δ8) and res-β-cat^Δ/Δ^ (rsc) mESC lines over a period of 5 days in feeder-free and serum-free culture (2i+LIF). Cell population doublings are plotted against days (n = 3, SD).
(TIF)

Figure S2 **Quantitative PCR examination of β-cat^fl/fl^, β-cat^fl/Δ^ and β-cat^Δ/Δ^ ESCs.** Expression levels of self-renewal marker genes (oct3/4, nanog, sox2 and klf4) relative to Gapdh in β-cat^fl/fl^ (blue bar: fl/fl1 and fl/fl2), β-cat^fl/Δ^ (purple bar: fl/Δ2 and fl/Δ3) and β-cat^Δ/Δ^ (light blue bar: Δ/Δ1, Δ/Δ2 and Δ/Δ8) mESCs.
(TIF)

Figure S3 **β-cat^Δ/Δ^ ESCs in serum- and feeder-free conditions of culture.** (A): Phase-contrast images of cellular expansion of β-cat^fl/fl^ and β-cat^Δ/Δ^ mESCs under serum- and feeder-free conditions using the 2i+LIF system with mitogen-activated protein kinase kinase (MEK) inhibitor (PD0325901) and GSK3β inhibitor (CHIR99021) on days 1, 3 and 5. Scale bars are 200 µm. (B): Quantitative PCR analysis of β-cat^fl/fl^ (fl/fl1 and fl/fl2) and β-cat^Δ/Δ^ (Δ/Δ1 and Δ/Δ2) mESCs in serum- and feeder-free conditions. Axin2 expression was normalized to Gapdh. In the canonical Wnt/β-catenin signaling cascade, Axin2 acts as the scaffold of the β-catenin destruction complex. Axin2 was not up-regulated in our β-cat^Δ/Δ^ mESCs, and so β-cat^Δ/Δ^ mESCs are transcriptionally defective in the canonical Wnt/β-catenin pathway.
(TIF)

Figure S4 **β-catenin-rescued β-cat^Δ/Δ^ ESCs showed restored development potential in the chimera assay.** (A): β-cat^Δ/Δ^ mESCs with an integrated piggyBac vector carrying a CAG promoter–driven β-catenin-2A-mCherry (res-β-cat^Δ/Δ^ mESCs) expressed red fluorescent protein mCherry. Scale bars are 500 µm. (B): Immunofluorescence staining for β-catenin (red), α-catenin (green), and E-cadherin (green) of res-β-cat^Δ/Δ^ mESC colonies as observed under confocal microscopy. Nuclei are stained for DAPI (blue). Scale bars are 20 µm. (C): Chimeras were generated by injection of res-β-cat^Δ/Δ^ mESCs into ICR host blastocysts. Chimeric embryos on E10.5 displayed the high contribution of res-β-cat^Δ/Δ^ mESCs to the whole body. Scale bars are 500 µm.
(TIF)

Figure S5 **Hierarchical clustering analysis of expression data from the TaqMan array across the 96 marker genes.** Multiple gene expression analysis of mESC lines and F9 (A) and tumors (B) by quantitative PCR using TaqMan Array Mouse Stem Cell Pluripotency Card (Life technologies). (A): The two subtypes of stem cell lines were clustered into distinct clusters with reversed gene expression patterns. The group of wild-type, res-β-cat^Δ/Δ^ and β-cat^Δ/Δ^ mESC lines was clustered from F9 EC. (B): Tumor clustering was different from stem cells. β-cat^Δ/Δ^ tumors were clustered into the same cluster as tumors derived from F9 EC, and separately clustered from teratomas of wild-type and res-β-cat^Δ/Δ^ mESCs. The level of expression of each gene in each sample, relative to the median level of expression of that gene across all the samples, is represented using a red-black-green color scale as shown in the key (green: below median; black: equal to median; red: above median).
(TIF)

Figure S6 **Chimeric embryos at E12.5 generated from EGFP-β-cat^Δ/Δ^ mESCs.** Contribution of EGFP-β-cat^Δ/Δ^ mESCs to mouse embryonic development. Embryos were analyzed using a ﬂuorescence stereomicroscope on E12.5. Embryos with scattered EGFP fluorescence showed limb malformations (white arrow head). Scale bars are 2 mm.
(TIF)

Figure S7 **Immunofluorescence staining of Plakoglobin in β-cat^fl/fl^, β-cat^Δ/Δ^ and res-β-cat** ^Δ/Δ^ **.** Immunofluorescence staining for Plakoglobin (green) and DAPI (blue) of β-cat^fl/fl^, β-cat^Δ/Δ^ and res-β-cat^Δ/Δ^ mESC colony as observed under confocal microscopy. Scale bars are 20 µm.
(TIF)

## References

[1] Marikawa Y (2006) Wnt/β-catenin signaling and body plan formation in mouse embryos. Semin Cell Dev Biol 17: 175–184. 16765611 10.1016/j.semcdb.2006.04.003

[2] MacDonald BT, Tamai K, He X (2009) Wnt/β-catenin signaling: Components, Mechanisms, and Diseases. Dev Cell 17: 9–26. 19619488 10.1016/j.devcel.2009.06.016 PMC2861485

[3] Evans MJ, Kaufman MH (1981) Establishment in culture of pluripotential cells from mouse embryos. Nature 292: 154–156. 7242681 10.1038/292154a0

[4] Thomson JA, Itskovitz-Eldor J, Shapiro SS, Waknitz MA, Swiergiel JJ, et al (1998) Embryonic stem cell lines derived from human blastocysts. Science 282: 1145–1147. 9804556 10.1126/science.282.5391.1145

[5] Sokol SY (2011) Maintaining embryonic stem cell pluripotency with Wnt signaling. Development 138: 4341–4350. 21903672 10.1242/dev.066209 PMC3177306

[6] Grigoryan T, Wend P, Klaus A, Birchmeier W (2008) Deciphering the function of canonical Wnt signals in development and disease: conditional loss- and gain-of-function mutations of beta-catenin in mice. Genes Dev 22: 2308–2341. 18765787 10.1101/gad.1686208 PMC2749675

[7] Lyashenko N, Winter M, Migliorini D, Biechele T, Moon RT, et al (2011) Differential requirement for the dual functions of β-catenin in embryonic stem cell self-renewal and germ layer formation. Nat Cell Biol 13: 753–761. 21685890 10.1038/ncb2260 PMC3130149

[8] Wray J, Kalkan T, Gomez-Lopez S, Eckardt D, Cook A, et al (2011) Inhibition of glycogen synthase kinase-3 alleviates Tcf3 repression of the pluripotency network and increases embryonic stem cell resistance to differentiation. Nat Cell Biol 13: 838–845. 21685889 10.1038/ncb2267 PMC3160487

[9] Takezawa Y, Yoshida K, Miyado K, Sato M, Nakamura A, et al (2011) β-catenin is a molecular switch that regulates transition of cell-cell adhesion to fusion. Sci Rep 1: 68. 22355587 10.1038/srep00068 PMC3216555

[10] Brault V, Moore R, Kutsch S, Ishibashi M, Rowitch DH, et al (2001) Inactivation of the beta-catenin gene by Wnt1-Cre-mediated deletion results in dramatic brain malformation and failure of craniofacial development. Development 128: 1253–1264. 11262227 10.1242/dev.128.8.1253

[11] Matsumura H, Hasuwa H, Inoue N, Ikawa M, Okabe M (2004) Lineage-specific cell disruption in living mice by Cre-mediated expression of diphtheria toxin A chain. Biochem Biophys Res Commun 321: 275–279. 15358172 10.1016/j.bbrc.2004.06.139

[12] Di Giorgio FP, Carrasco MA, Siao MC, Maniatis T, Eggan K (2007) Non-cell autonomous effect of glia on motor neurons in an embryonic stem cell-based ALS model. Nat Neurosci 10: 608–614. 17435754 10.1038/nn1885 PMC3139463

[13] Ying QL, Wray J, Nichols J, Batlle-Morera L, Doble B, et al (2008) The ground state of embryonic stem cell self-renewal. Nature 453: 519–523. 18497825 10.1038/nature06968 PMC5328678

[14] Silva J, Barrandon O, Nichols J, Kawaguchi J, Theunissen TW, Smith A (2008) Promotion of reprogramming to ground state pluripotency by signal inhibition. PLoS Biol 6: e253. 18942890 10.1371/journal.pbio.0060253 PMC2570424

[15] Akutsu H, Miura T, Machida M, Birumachi J, Hamada A, et al (2009) Maintenance of pluripotency and self-renewal ability of mouse embryonic stem cells in the absence of tetraspanin CD9. Differentiation 78: 137–142. 19716222 10.1016/j.diff.2009.08.005

[16] Nagy A, Rossant J, Nagy R, Abramow-Newerly W, Roder JC (1993) Derivation of completely cell-culture derived mice from early-passage embryonic stem cells. Proc Natl Acad Sci USA 90: 8424–8428. 8378314 10.1073/pnas.90.18.8424 PMC47369

[17] Yusa K, Rad R, Takeda J, Bradley A (2009) Generation of transgene-free induced pluripotent mouse stem cells by the *piggyBac* transposon. Nat Methods 6: 363–369. 19337237 10.1038/nmeth.1323 PMC2677165

[18] Ishii R, Kami D, Toyoda M, Makino H, Gojo S, et al (2012) Placenta to Cartilage: Direct conversion of human placenta to chondrocytes with transformation by defined factors. Mol Biol Cell 23: 3511–3521. 22833560 10.1091/mbc.E11-10-0869 PMC3442400

[19] Ikawa M, Yamada S, Nakanishi T, Okabe M (1999) Green fluorescent protein (GFP) as a vital marker in mammals. Curr Top Dev Biol 44: 1–20. 9891875 10.1016/s0070-2153(08)60465-2

[20] Stadtfed M, Apostolou E, Akutsu H, Fukuda A, Follett P, et al (2010) Aberrant silencing of imprinted genes on chromosome 12qF1 in mouse induced stem cell. Nature 465: 175–181. 20418860 10.1038/nature09017 PMC3987905

[21] Haegel H, Larue L, Ohsugi M, Fedorov L, Herrenknecht K, et al (1995) Lack of beta-catenin affects mouse development at gastrulation. Development 121: 3529–3537. 8582267 10.1242/dev.121.11.3529

[22] Huelsken J, Vogel R, Brinkmann V, Erdmann B, Birchmeier C, et al (2000) Requirement for beta-catenin in anterior-posterior axis formation in mice. J Cell Biol 148: 567–578. 10662781 10.1083/jcb.148.3.567 PMC2174807

[23] Arnold SJ, Stappert J, Bauer A, Kispert A, Herrmann BG, et al (2000) Brachyury is a target gene of the Wnt/beta-catenin signaling pathway. Mech Dev 91: 249–258. 10704849 10.1016/s0925-4773(99)00309-3

[24] Eble JN, Sauter G, Epstein JI, Sesterhenn IA (2004) Pathology and genetics of tumours of the urinary system and male genital organs. Lyon, France: IARC Press: 218–249. World Health Organization Classification of Tumours.

[25] Oosterhuis JW, Looijenga LH (2005) Testicular germ-cell tumours in a broader perspective. Nat Rev Cancer 5: 210–222. 15738984 10.1038/nrc1568

[26] Bahrami A, Ro JY, Ayala AG (2007) An overview of testicular germ cell tumors. Arch Pathol Lab Med 131: 1267–1279. 17683189 10.5858/2007-131-1267-AOOTGC

[27] Talerman A (1985) Germ cell tumours. Ann Pathol 5: 145–157. 3000396

[28] Cao D, Humphrey PA, Allan RW (2009) SALL4 is a novel sensitive and specific marker for metastatic germ cell tumors, with particular utility in detection of metastatic yolk sac tumors. Cancer 115: 2640–2651. 19365862 10.1002/cncr.24308

[29] Cao D, Li J, Guo CC, Allan RW, Humphrey PA (2009) SALL4 is a novel diagnostic marker for testicular germ cell tumors. Am J Surg Pathol 33: 1065–1077. 19390421 10.1097/PAS.0b013e3181a13eef

[30] Talerman A, Haije WG, Baggerman L (1980) Serum alpharfetoprotein (AFP) in patients with germ cell tumors of the gonads and extragonadal sites: correlation between endodermal sinus (yolk sac) tumor and raised serum AFP. Cancer 46: 380–385. 6155988 10.1002/1097-0142(19800715)46:2<380::aid-cncr2820460228>3.0.co;2-u

[31] Boiani M, Scholer HR (2005) Regulatory networks in embryo-derived pluripotent stem cells. Nat Rev Mol Cell Biol 6: 872–884. 16227977 10.1038/nrm1744

[32] Beachy PA, Karhadkar SS, Berman DM (2004) Tissue repair and stem cell renewal in carcinogenesis. Nature 432: 324–331. 15549094 10.1038/nature03100

[33] Laird DJ, von Andrian UH, Wagers AJ (2008) Stem cell trafficking in tissue development, growth, and disease. Cell 132: 612–630. 18295579 10.1016/j.cell.2008.01.041

[34] Murry CE, Keller G (2008) Differentiation of embryonic stem cells to clinically relevant populations: lessons from embryonic development. Cell 132: 661–680. 18295582 10.1016/j.cell.2008.02.008

[35] Niwa H (2007) How is pluripotency determined and maintained? Development 134: 635–646. 17215298 10.1242/dev.02787

[36] Davidson KC, Adams AM, Goodson JM, McDonald CE, Potter JC, et al (2012) Wnt/β-catenin signaling promotes differentiation, not self-renewal, of human embryonic stem cells and is repressed by Oct4. Proc Natl Acad Sci U S A 109: 4485–4490. 22392999 10.1073/pnas.1118777109 PMC3311359

[37] DeVries WN, Evsikov AV, Haac BE, Fancher KS, Holbrook AE, et al (2004) Maternal beta-catenin and E-cadherin in mouse development. Development 131: 4435–4445. 15306566 10.1242/dev.01316

[38] Xie H, Tranguch S, Jia X, Zhang H, Das SK, et al (2008) Inactivation of nuclear Wnt-β-catenin signaling limits blastocyst competency for implantation. Development 135: 717–727. 18199579 10.1242/dev.015339 PMC2829274

[39] Clevers H (2006) Wnt/beta-catenin signaling in development and disease. Cell 127: 469–480. 17081971 10.1016/j.cell.2006.10.018

[40] Moon RT, Kohn AD, De Ferrari GV, Kaykas A (2004) WNT and beta-catenin signalling: diseases and therapies. Nat Rev Genet 5: 691–701. 15372092 10.1038/nrg1427

[41] Clevers H, Nusse R (2012) Wnt/β-catenin Signaling and Disease. Cell 149: 1192–1205. 22682243 10.1016/j.cell.2012.05.012

[42] Reya T, Clevers H (2005) Wnt signaling in stem cells and cancer. Nature 434: 843–850. 15829953 10.1038/nature03319

[43] Kielman MF, Rindapää M, Gaspar C, van Poppel N, Breukel C, et al (2002) Apc modulates embryonic stem-cell differentiation by controlling the dosage of beta-catenin signaling. Nat Genet 32: 594–605. 12426568 10.1038/ng1045

[44] Doble BW, Patel S, Wood GA, Kockeritz LK, Woodgett JR (2007) Functional redundancy of GSK-3alpha and GSK-3beta in Wnt/beta-catenin signaling shown by using an allelic series of embryonic stem cell lines. Dev Cell 12: 957–971. 17543867 10.1016/j.devcel.2007.04.001 PMC4485918

