# Supplementary figures and images for: β-Catenin Functions Pleiotropically in Differentiation and Tumorigenesis in Mouse Embryo-Derived Stem Cells
Source: PLoS One. 2013 May 14;8(5):e63265. doi: 10.1371/journal.pone.0063265 (PMC3653942; doi:10.1371/journal.pone.0063265)

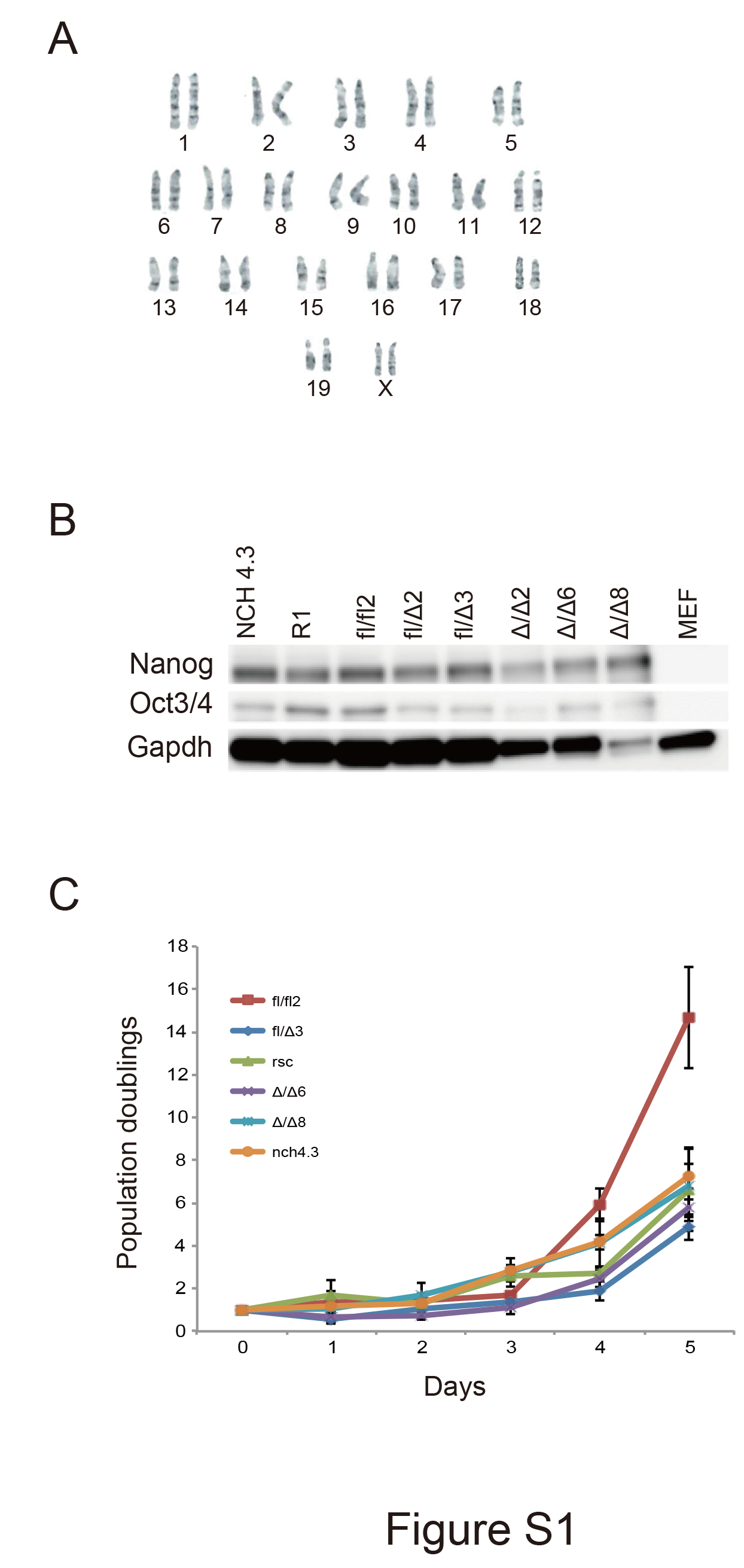

Supplement: Figure S1 — Growth curve, western blotting and chromosome analysis of embryo-derived β-catΔ/Δ mESCs. (A): The examined β-catΔ/Δ mESC lines had normal karyotypes. The β-catΔ/Δ (Δ/Δ2) mESC line showed a normal karyotype of 40XX. (B): Western blots of Nanog and Oct3/4 in wild-type (NCH4.3 and R1), β-catfl/fl (fl/fl2), β-catfl/Δ (fl/Δ2 and fl/Δ3), β-catΔ/Δ (Δ/Δ2, Δ/Δ6 and Δ/Δ8) mESCs and MEF. (C): Growth curve of β-catfl/fl (fl/fl2), β-catfl/Δ (fl/Δ3), β-catΔ/Δ (Δ/Δ6 and Δ/Δ8) and res-β-catΔ/Δ (rsc) mESC lines over a period of 5 days in feeder-free and serum-free culture (2i+LIF). Cell population doublings are plotted against days (n = 3, SD). (TIF) [file pone.0063265.s001.tif]

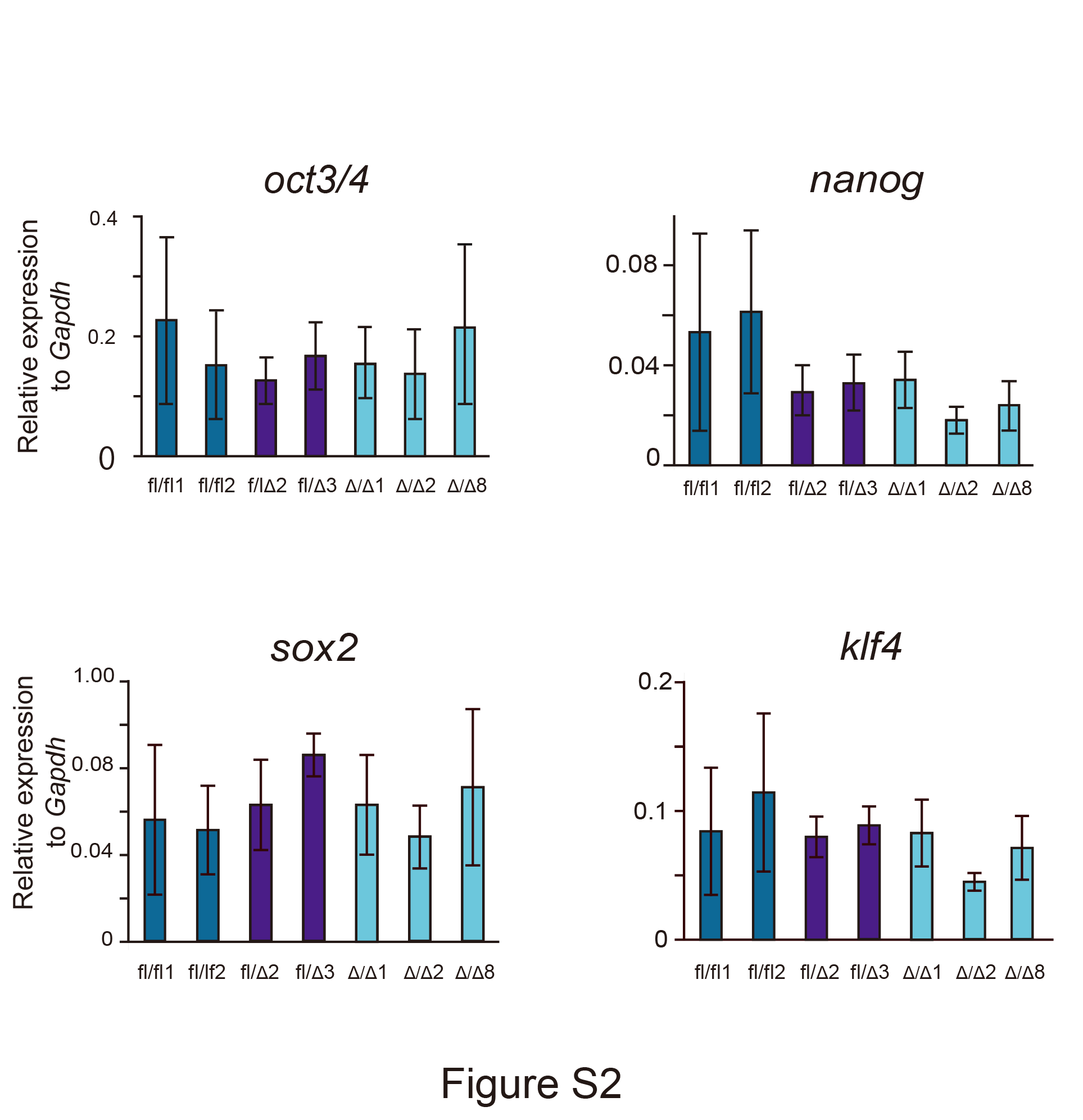

Supplement: Figure S2 — Quantitative PCR examination of β-catfl/fl, β-catfl/Δ and β-catΔ/Δ ESCs. Expression levels of self-renewal marker genes (oct3/4, nanog, sox2 and klf4) relative to Gapdh in β-catfl/fl (blue bar: fl/fl1 and fl/fl2), β-catfl/Δ (purple bar: fl/Δ2 and fl/Δ3) and β-catΔ/Δ (light blue bar: Δ/Δ1, Δ/Δ2 and Δ/Δ8) mESCs. (TIF) [file pone.0063265.s002.tif]

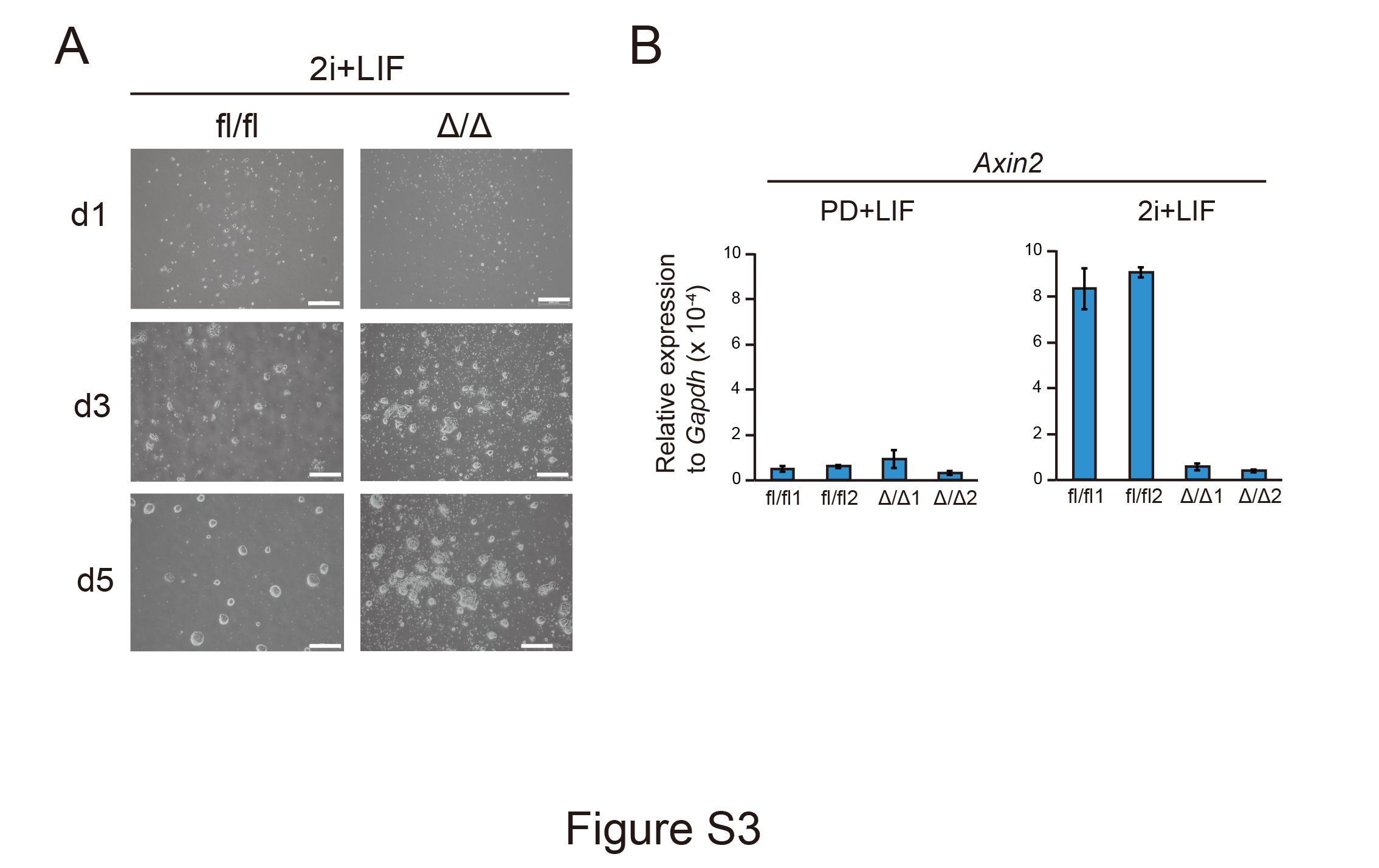

Supplement: Figure S3 — β-catΔ/Δ ESCs in serum- and feeder-free conditions of culture. (A): Phase-contrast images of cellular expansion of β-catfl/fl and β-catΔ/Δ mESCs under serum- and feeder-free conditions using the 2i+LIF system with mitogen-activated protein kinase kinase (MEK) inhibitor (PD0325901) and GSK3β inhibitor (CHIR99021) on days 1, 3 and 5. Scale bars are 200 µm. (B): Quantitative PCR analysis of β-catfl/fl (fl/fl1 and fl/fl2) and β-catΔ/Δ (Δ/Δ1 and Δ/Δ2) mESCs in serum- and feeder-free conditions. Axin2 expression was normalized to Gapdh. In the canonical Wnt/β-catenin signaling cascade, Axin2 acts as the scaffold of the β-catenin destruction complex. Axin2 was not up-regulated in our β-catΔ/Δ mESCs, and so β-catΔ/Δ mESCs are transcriptionally defective in the canonical Wnt/β-catenin pathway. (TIF) [file pone.0063265.s003.tif]

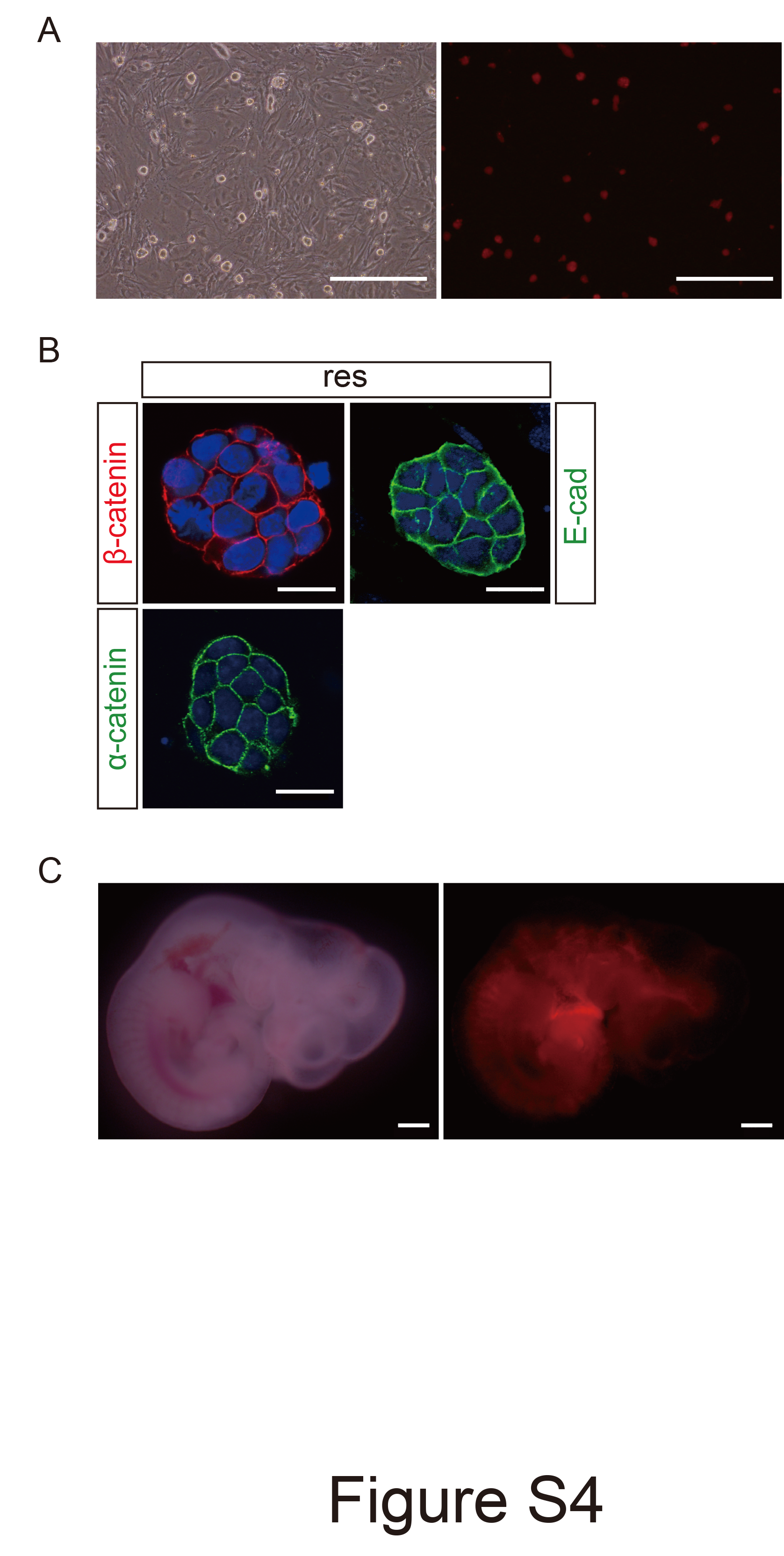

Supplement: Figure S4 — β-catenin-rescued β-catΔ/Δ ESCs showed restored development potential in the chimera assay. (A): β-catΔ/Δ mESCs with an integrated piggyBac vector carrying a CAG promoter–driven β-catenin-2A-mCherry (res-β-catΔ/Δ mESCs) expressed red fluorescent protein mCherry. Scale bars are 500 µm. (B): Immunofluorescence staining for β-catenin (red), α-catenin (green), and E-cadherin (green) of res-β-catΔ/Δ mESC colonies as observed under confocal microscopy. Nuclei are stained for DAPI (blue). Scale bars are 20 µm. (C): Chimeras were generated by injection of res-β-catΔ/Δ mESCs into ICR host blastocysts. Chimeric embryos on E10.5 displayed the high contribution of res-β-catΔ/Δ mESCs to the whole body. Scale bars are 500 µm. (TIF) [file pone.0063265.s004.tif]

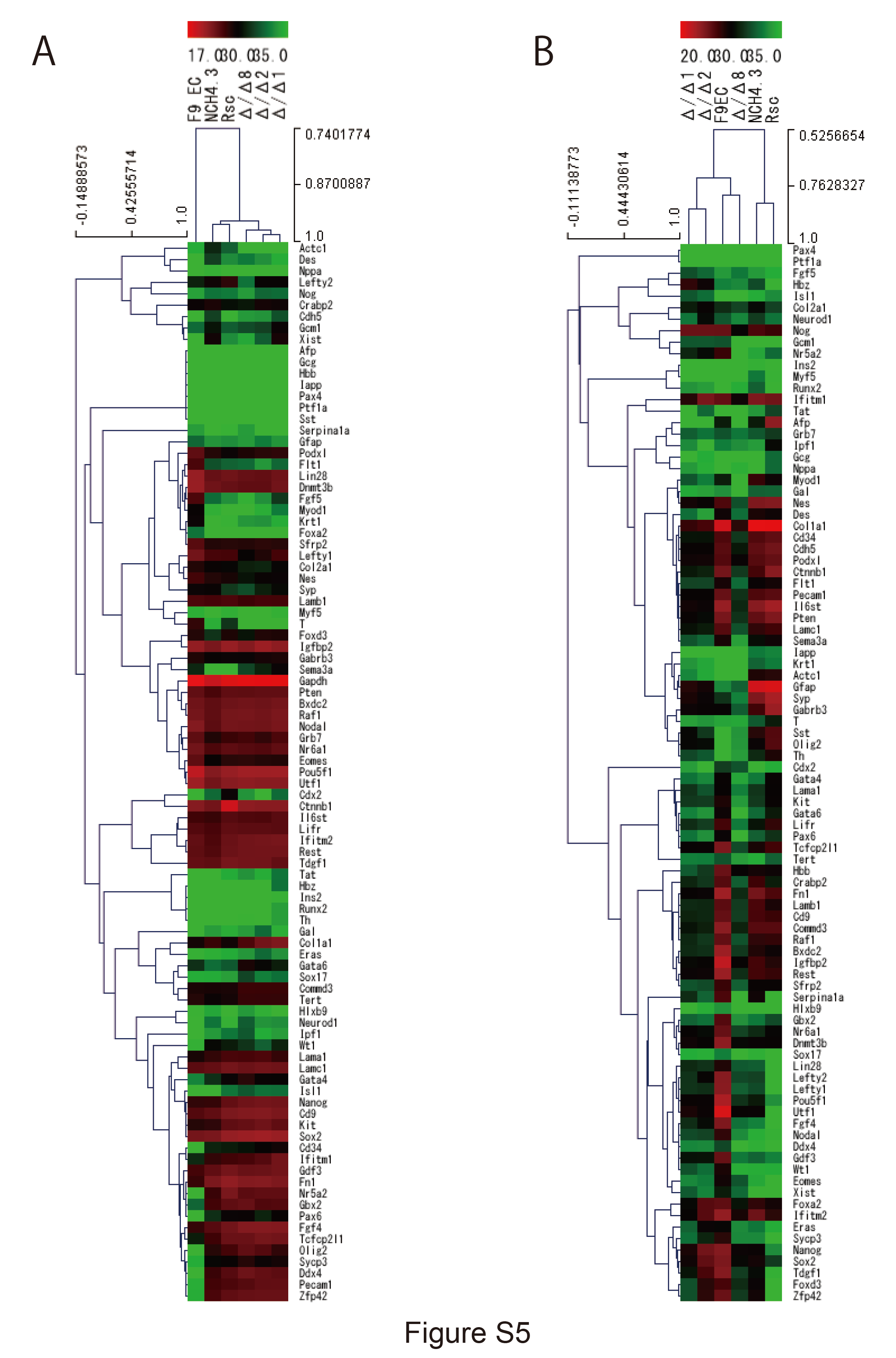

Supplement: Figure S5 — Hierarchical clustering analysis of expression data from the TaqMan array across the 96 marker genes. Multiple gene expression analysis of mESC lines and F9 (A) and tumors (B) by quantitative PCR using TaqMan Array Mouse Stem Cell Pluripotency Card (Life technologies). (A): The two subtypes of stem cell lines were clustered into distinct clusters with reversed gene expression patterns. The group of wild-type, res-β-catΔ/Δ and β-catΔ/Δ mESC lines was clustered from F9 EC. (B): Tumor clustering was different from stem cells. β-catΔ/Δ tumors were clustered into the same cluster as tumors derived from F9 EC, and separately clustered from teratomas of wild-type and res-β-catΔ/Δ mESCs. The level of expression of each gene in each sample, relative to the median level of expression of that gene across all the samples, is represented using a red-black-green color scale as shown in the key (green: below median; black: equal to median; red: above median). (TIF) [file pone.0063265.s005.tif]

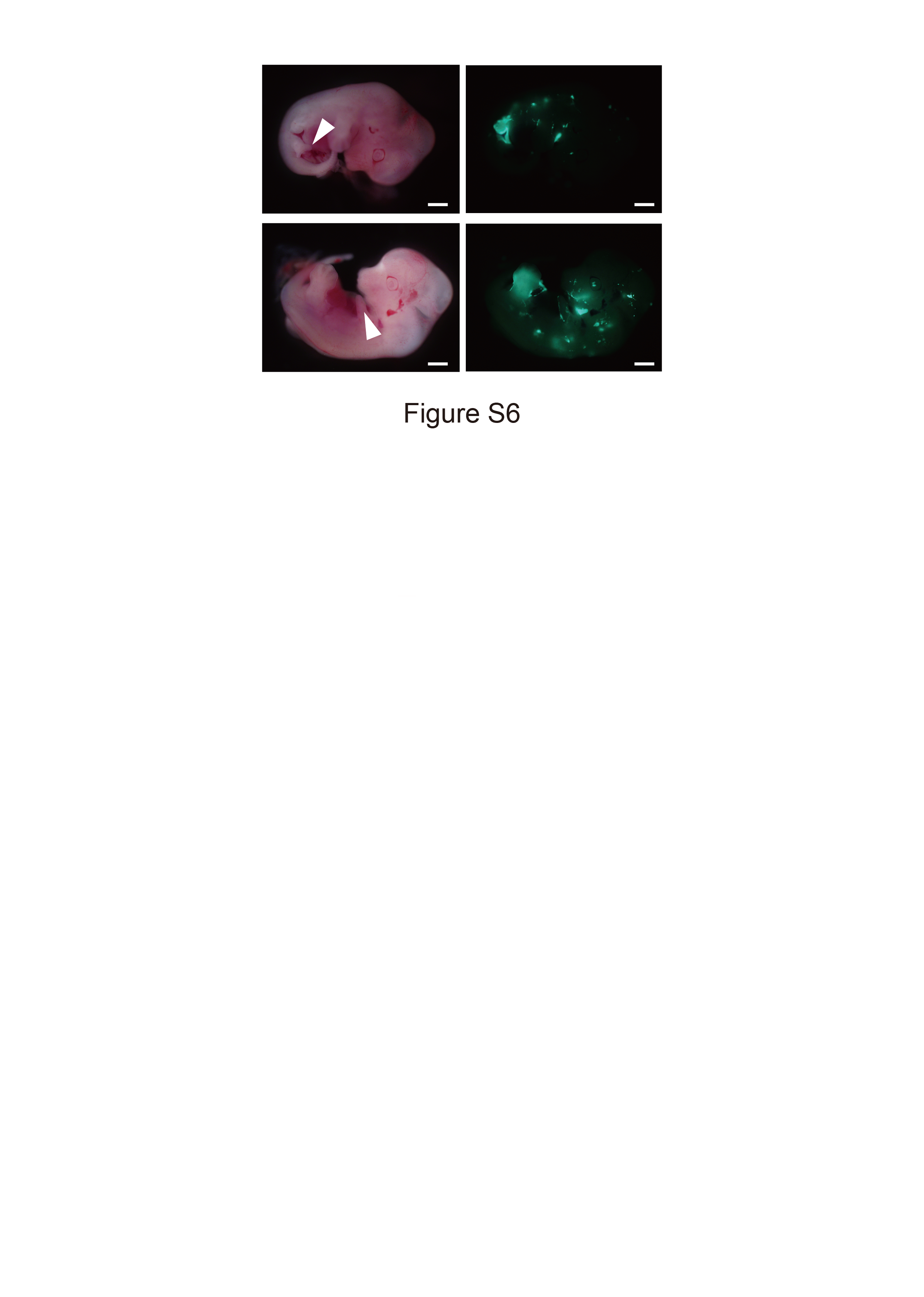

Supplement: Figure S6 — Chimeric embryos at E12.5 generated from EGFP-β-catΔ/Δ mESCs. Contribution of EGFP-β-catΔ/Δ mESCs to mouse embryonic development. Embryos were analyzed using a ﬂuorescence stereomicroscope on E12.5. Embryos with scattered EGFP fluorescence showed limb malformations (white arrow head). Scale bars are 2 mm. (TIF) [file pone.0063265.s006.tif]

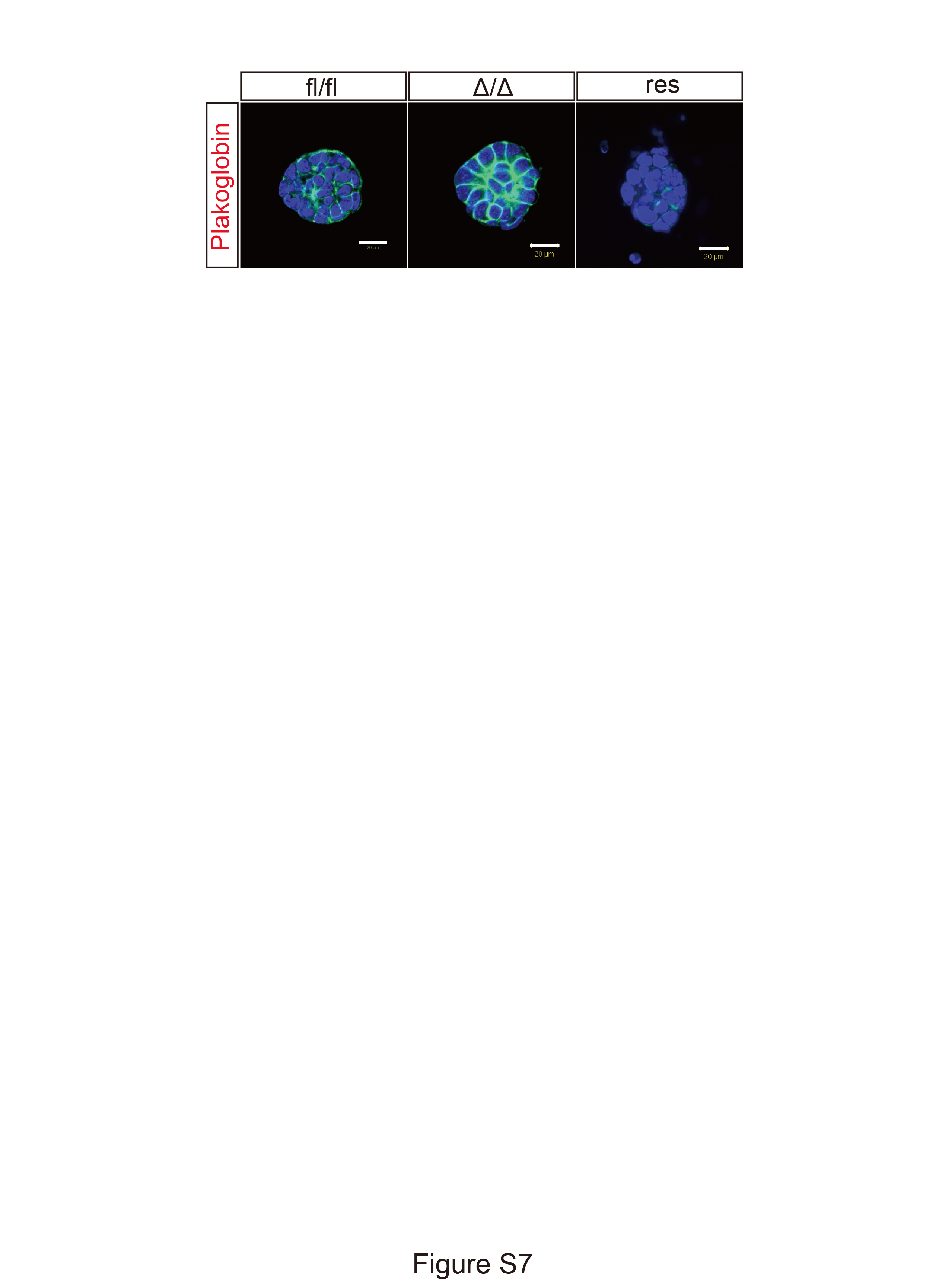

Supplement: Figure S7 — Immunofluorescence staining of Plakoglobin in β-catfl/fl, β-catΔ/Δ and res-β-cat Δ/Δ . Immunofluorescence staining for Plakoglobin (green) and DAPI (blue) of β-catfl/fl, β-catΔ/Δ and res-β-catΔ/Δ mESC colony as observed under confocal microscopy. Scale bars are 20 µm. (TIF) [file pone.0063265.s007.tif]
